# The human discs large protein 1 interacts with and maintains connexin 43 at the plasma membrane in keratinocytes

**DOI:** 10.1242/jcs.259984

**Published:** 2023-06-08

**Authors:** Harry Scott, Li Dong, Andrew Stevenson, Alasdair I. MacDonald, Sharmila Srinivasan, Paola Massimi, Lawrence Banks, Patricia E. Martin, Scott R. Johnstone, Sheila V. Graham

**Affiliations:** ^1^ MRC-University of Glasgow Centre for Virus Research, School of Infection and Immunity, College of Medical Veterinary and Life Sciences, University of Glasgow, Garscube Estate, Glasgow G61 1QH, UK; ^2^ Translation Research Platform for Veterinary Biologicals, Chennai, Tamil Nadu, India; ^3^ International Centre for Genetic Engineering and Biotechnology, Trieste, Italy; ^4^ Department of Biological and Biomedical Sciences, School of Health and Life Sciences, Glasgow Caledonian University, Glasgow G4 0BA, UK; ^5^ Fralin Biomedical Research Institute at Virginia Tech Carilion, Center for Vascular and Heart Research, Virginia Tech, Roanoke VA 24016, USA

**Keywords:** Connexin 43, MAGUK proteins, Dlg1, Keratinocytes, Gap junctional communication

## Abstract

Gap junction channels, composed of connexins, allow direct cell-to-cell communication. Connexin 43 (Cx43; also known as GJA1) is widely expressed in tissues, including the epidermis. In a previous study of human papillomavirus-positive cervical epithelial tumour cells, we identified Cx43 as a binding partner of the human homologue of *Drosophila* Discs large (Dlg1; also known as SAP97). Dlg1 is a member of the membrane associated-guanylate kinase (MAGUK) scaffolding protein family, which is known to control cell shape and polarity. Here, we show that Cx43 also interacts with Dlg1 in uninfected keratinocytes *in vitro* and in keratinocytes, dermal cells and adipocytes in normal human epidermis *in vivo*. Depletion of Dlg1 in keratinocytes did not alter Cx43 transcription but was associated with a reduction in Cx43 protein levels. Reduced Dlg1 levels in keratinocytes resulted in a reduction in Cx43 at the plasma membrane with a concomitant reduction in gap junctional intercellular communication and relocation of Cx43 to the Golgi compartment. Our data suggest a key role for Dlg1 in maintaining Cx43 at the plasma membrane in keratinocytes.

## Teaser

**Summary:** Discs large protein 1 (Dlg1) interacts with the gap junction protein connexin 43 (Cx43) in keratinocytes. Dlg1 depletion leads to low Cx43 levels and reduced intercellular communication. Dlg1 might maintain Cx43 at the plasma membrane.

## INTRODUCTION

Intercellular communication via gap junction channels is critical for multiple physiological processes including normal tissue function. For epithelial tissues, gap junctions maintain epithelial integrity and are involved in epidermal innate immunity (Chanson et al., 2018; Evans, 2015; Valdebenito et al., 2018). As a major component of intercellular signalling, gap junctions permit cell-to-cell transfer of ions, metabolites and small nucleic acids (<1 kDa) to control cellular functions such as migration and proliferation (Laird, 2006). Gap junctions are formed when two hexameric channels, composed of connexin (Cx) proteins, in the plasma membrane of opposing cells, dock to permit direct cell-to-cell communication (Aasen et al., 2019). Connexin 43 (Cx43; also known as GJA1) is the most ubiquitously expressed connexin with widespread tissue distribution. Importantly, it has key roles in skin physiology (Lilly et al., 2016; Martin et al., 2014), and changes in its localisation and post-translational modification have been implicated in epithelial wound healing pathologies (Lorraine et al., 2015). The life cycle of Cx43 is brief (∼1.5 h) and involves trafficking from the endoplasmic reticulum (ER)/Golgi (Musil and Goodenough, 1993) to the plasma membrane via microtubules and associated motor proteins (Shaw et al., 2007) for gap junction assembly. Cx43 can be internalised, probably following ubiquitylation (Leithe and Rivedal, 2004), as part of hemichannels or gap junction channels. Connexins are rapidly recycled through the autophagosomal/lysosomal proteasomal pathways (Berthoud et al., 2004; Epifantseva and Shaw, 2018; Falk et al., 2014; Su and Lau, 2014; Totland et al., 2020). This delicate balance of trafficking, assembly and recycling is facilitated by dynamic protein–protein interactions with trafficking partners and is highly susceptible to changes in the cellular environment (Aasen et al., 2018; Johnstone et al., 2012; Leithe et al., 2018).

Multiple interacting partners have been associated with alterations in the trafficking and turnover of full-length Cx43 (Aasen et al., 2018; Leithe et al., 2018; Su and Lau, 2014). Recently, it has been found that internal translation sites for Cx43 can produce smaller Cx43 protein fragments that might exert direct control over full-length Cx43 protein trafficking (Salat-Canela et al., 2014; Zeitz et al., 2019). Interactions with structural proteins, including tubulin (Basu et al., 2017; Giepmans et al., 2001; Kang et al., 2009; Lauf et al., 2002; Saidi Brikci-Nigassa et al., 2012; Shaw et al., 2007) and actin (Meng and Yan, 2020; Smyth et al., 2012; Theiss and Meller, 2002; Thomas et al., 2001), are associated with control of plasma membrane trafficking. Other molecular chaperone proteins can link Cx43 to the cytoskeleton; for example, through its N-terminus, drebrin can bind the Cx43 C-terminus, forming a link to the actin cytoskeleton (Ambrosi et al., 2016; Butkevich et al., 2004; Palatinus et al., 2012). The most studied Cx43 interaction is that with zonula occludens-1 (ZO-1; also known as TJP1), a plasma membrane associated-guanylate kinase (MAGUK) family multi-domain protein (Ambrosi et al., 2016; Gourdie et al., 2006; Hunter et al., 2005; Rhett et al., 2010; Sorgen et al., 2004; Zheng et al., 2019). ZO-1 binds directly to the terminal five amino acid residues of Cx43 in a tubulin-linked super-complex to regulate channel insertion into the plasma membrane, gap junction channel aggregation in plaques and recycling of old gap junctions from the plaque (Giepmans and Moolenar, 1998; Palatinus et al., 2012; Rhett et al., 2010; Solan and Lampe, 2014; Toyofuku et al., 1998).

MAGUK proteins, which have roles in cell signalling cascades and cell morphology organisation (Subbaiah et al., 2011; Won et al., 2017; Ye et al., 2018), can link cell junctions to cell shape and cell signalling. The mammalian homologue of *Drosophila* discs large protein (Dlg1; also known as SAP97) is a MAGUK protein, which like ZO-1, is expressed in epithelial tissues. Dlg1 is found predominantly at epithelial intercellular contact sites in adherens junctions where it binds E-cadherin to link to α- and β-catenins and the actin cytoskeleton (Firestein and Rongo, 2001; Reuver and Garner, 1998; Wu et al., 1998). These interactions are critical components of epithelial junctional integrity and barrier function (Laprise et al., 2004; Reuver and Garner, 1998). Dlg1 is part of the Scribble complex (Stephens et al., 2018), which maintains epithelial cell architecture and polarity and represses cell proliferation (Bonilha and Rodriguez-Boulan, 2001; Golub et al., 2017; Knoblich, 2008; Müller et al., 1995; O'Neill et al., 2011; Woods et al., 1996). Inactivation by viral oncoproteins is linked to the development of epithelial-derived human cancers, such as cervical cancer (James and Roberts, 2016; Subbaiah et al., 2011).

Dlg1 was originally identified as a Cx43-binding protein in a tandem mass spectrometry analysis of normal rat kidney cell lysates (Singh and Lampe, 2003). It was subsequently shown to bind to connexin 32 (Cx32) through its C-terminal GUK domain (Duffy et al., 2007; Stauch et al., 2012). Previously, we have demonstrated that Cx43 can bind Dlg1 in human papillomavirus (HPV)-positive cervical tumour cells. We showed that the human papillomavirus (HPV) E6 oncoprotein, through its interaction with Dlg1, can sequester Cx43 in a cytoplasmic location and inhibit gap junctional communication (MacDonald et al., 2012; Sun et al., 2015).

Although these studies clearly defined a role for a Dlg1–Cx43 interaction in HPV-positive cancer cells, interaction in other cell types and in the absence of the HPV oncoprotein has not been investigated. Cx43 and Dlg1 have been found to interact directly *in vitro* (MacDonald et al., 2012) suggesting that they might interact in non-pathological situations. Here we demonstrate that the Dlg1–Cx43 interaction occurs in a range of cell types in non-cancerous human cutaneous tissue and in cultured keratinocytes. The Dlg1–Cx43 interaction is functionally relevant because Dlg1 knockdown in HaCaT keratinocytes resulted in reduced Cx43 protein levels. When Dlg1 was depleted in cells, some Cx43 colocalised with markers of the Golgi. Coincident with this, depletion of Dlg1 inhibited gap junctional intercellular communication. Taken together, our data suggests that interaction between Cx43 and Dlg1 is important in maintaining Cx43 at the plasma membrane in non-cancerous epithelial cells.

## RESULTS

### Cx43 and Dlg1 colocalise *in vivo* and *in vitro*

**Fig. 1.**
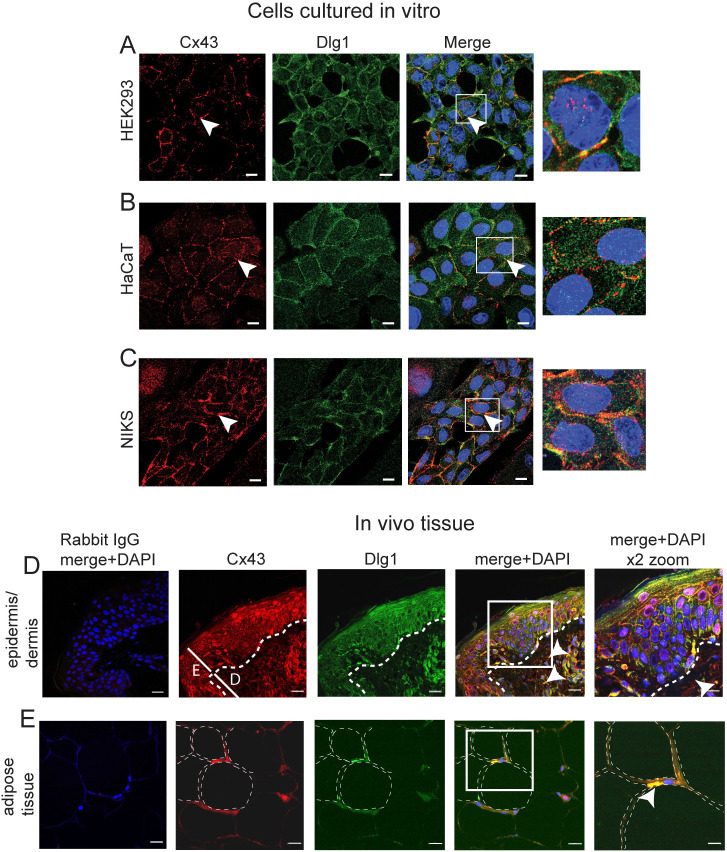
**Cx43 and Dlg1 colocalise in keratinocytes *in vitro* and *in vivo*.** (A–C) Cx43 (red staining) and Dlg1 (green staining) localisation in (A) HEK293 cells, (B) HaCaT cells and (C) NIKS cells. Blue staining, DAPI-stained nuclei. White arrowheads indicate plasma membrane colocalisation of Cx43 and Dlg1. Scale bars: 10 µm. The boxed areas in the merged images are shown as enlarged images to the right-hand side. These images are representative of five separate experiments. (D) Cx43 (red) Dlg1 (green) and DAPI (blue) staining of an epidermal skin tissue section representative of tissues from three individuals. A negative control staining with rabbit IgG is shown to the left-hand side. Dotted white lines indicate the basal layer of the epithelium. White lines mark the, E, epidermis, and, D, dermis layers. White arrowheads indicate Cx43 and Dlg1 colocalisation in the dermis. A Zen Zeiss microscopy digital 2× zoom image is shown to the right-hand side. Similar images were obtained from three separate tissues. (E) Cx43 (red staining) colocalisation with Dlg1 (green staining) in adipocytes in the tissue section. Nuclei are stained with DAPI. Fat deposits in the cytoplasm of selected cells are outlined with dotted lines. All images representative of at least three repeats. Scale bars: 20 µm (main images in D, E); 10 µm (2× zoom images in D, E).

Previous studies from our laboratory have demonstrated that there is a close association of Cx43 and Dlg1 in cultured tumour cells of a keratinocyte lineage and a direct interaction of the purified proteins *in vitro* (MacDonald et al., 2012; Sun et al., 2015). However, whether these two proteins interact *in vivo*, in non-tumour epithelial cells, has never been addressed. First we examined Dlg1–Cx43 location *in vitro* using HaCaT (spontaneously immortalised skin keratinocytes; Boukamp et al., 1988) and NIKS (near-diploid, spontaneously immortalised foreskin keratinocytes; Allen-Hoffmann et al., 2000) cells, as well as HEK293 cells, which are thought to be derived from human kidney epithelial cells. In confluent areas of HEK293 cells, Cx43 primarily localized to the borders of cells, with staining consistent with gap junction plaques (arrowhead) but some Cx43 was also located in the cytoplasm and the nucleus (Fig. 1A, left panel). In these cells, although Dlg1 exhibited a more disperse intracellular location than Cx43, the proteins showed colocalisation at the plasma membrane (Fig. 1A). The pattern of Cx43 location was similar in HaCaT cells and NIKS cells, with mostly plasma membrane location and some intracytoplasmic and nuclear staining (highlighted, boxed areas). There was significant colocalisation with Dlg1 at the cell periphery (Fig. 1B,C arrowheads, merge panels).

Next, we investigated Cx43 and Dlg1 expression and interaction in normal healthy tissues (abdominal skin biopsies) from three individuals (Fig. 1D). Similar data were obtained in each case. The location of Cx43 and Dlg1 in the tissue was visualised by confocal microscopy (Fig. 1D). Cx43 (red staining) and Dlg1 (green staining) was expressed throughout the epithelial layers of the tissue and colocalisation was observed at the cell periphery (Fig. 1D, merge+DAPI). Some colocalisation was also present in some cells in the dermis (white arrowheads) suggesting that Cx43 interacts with Dlg1 in cells other than epithelial cells. The tissue sections also contained adipose tissue (Fig. 1E), in which Cx43 is expressed (Kim et al., 2017). Colocalisation of Cx43 with Dlg1 was also apparent at plasma cell membranes in adipocytes (Fig. 1E, merge+DAPI ×2 zoom, white arrowhead). No staining was observed in the epithelium or in the adipose tissue using an IgG negative control. These data indicate that Cx43 colocalises with Dlg1 in several different cell types in cutaneous tissue.

### Cx43 forms a protein complex with Dlg1 in epithelial cells

**Fig. 2.**
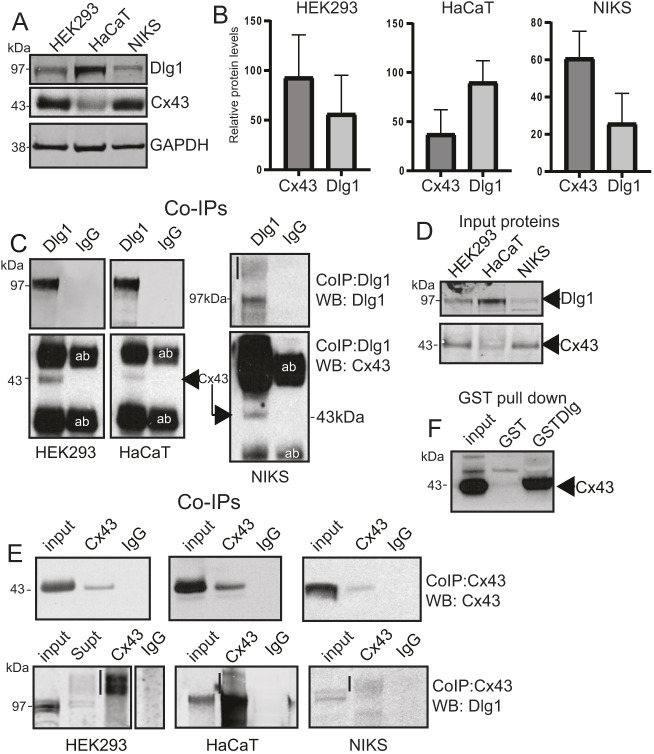
**Cx43 and Dlg1 co-immunoprecipitation.** (A) Western blot analysis of Cx43 and Dlg1 in HEK293, HaCaT and NIKS cells. (B) Quantification of protein levels in each cell line relative to the GAPDH loading control showing the mean±s.d. of three separate experiments. (C) Top panels, co-immunoprecipitation of endogenous Dlg1 by Dlg1 antibody H60 in each keratinocyte line. Bottom panels, co-immunoprecipitation of endogenous Cx43 using the H60 anti-Dlg1 antibody. IgG, control immunoprecipitation using an antibody of the same isotype. The heavy bands above and below the Cx43 band are antibody heavy and light chains (labelled ‘ab’). Vertical black line indicates multimerised Dlg1. (D) Western blots of input proteins: 10% of the amount of cell extract used in the co-immunoprecipitation assays. (E) Top panels, co-immunoprecipitation of endogenous Cx43 from the three cell types using an anti-Cx43 antibody C-6219 (Sigma). Bottom panels, co-immunoprecipitation of endogenous Dlg1 from the three cell types using the anti-Cx43 antibody. IgG, control immunoprecipitation using an antibody of the same isotype. An extra lane on the lower western blot for HEK293 cells shows remaining unprecipitated Dlg1 in the supernatant from the co-immunoprecipitation experiment. Vertical black lines indicate multimerised Dlg1. (F) GST pulldown experiment in HaCaT cells showing that GST–Dlg1 can interact with endogenous Cx43. All images representative of at least three repeats.

Western blot analysis in HaCaT, NIKS and HEK293 cells revealed that all cells expressed Dlg1 and Cx43 proteins, although differences were observed in the relative proportions of Cx43 and Dlg1 levels between the cell types (Fig. 2A,B). Cx43 is normally associated with the lower layers, which comprise less-differentiated epithelial cells (O'Shaughnessy et al., 2021), therefore these differences might reflect the capacity for differentiation of the individual cell types. Co-immunoprecipitation experiments showed that anti-Dlg1 antibody could immunoprecipitate Dlg1 and Cx43 from cell lysates of HaCaT, HEK293 and NIKS cells (Fig. 2C). In the western blots presented in the lower panel reacted with the Cx43 antibody, the prominent upper and lower bands are antibody heavy and light chains. There is only one antibody against Dlg1 (H60) that can be used successfully in co-immunoprecipitation but a large amount of immunoglobulin is co-eluted and detected by the Cx43 antibody (MacDonald et al., 2012). Input proteins are shown on a separate western blot, which was carried out at the same time as the co-immunoprecipitation blots (Fig. 2D). A separate blot was necessary since the input bands were not sufficiently visible on the co-immunoprecipitation blots due to the density of the antibody bands, precluding longer exposure of the blots. In a reverse reaction, anti-Cx43 antibody was able to immunoprecipitate Cx43 and Dlg1 from the three cell types (Fig. 2E). Dlg1 multimerised in the presence of Cx43, particularly in HEK293 cells, as has been shown previously (Marfatia et al., 2000; Nakagawa et al., 2004). Dlg1 can homo- or hetero-multimerise through its L27 domain. Multimerised Dlg1 is indicated with vertical black lines (Fig. 2C,E).

Previously we have shown that Cx43 could be detected as binding to Dlg1 in a GST pulldown experiment in cervical cancer cells and that GST–Dlg1 could interact directly *in vitro* with FLAG-tagged Cx43 (MacDonald et al., 2012). Fig. 2F shows that GST–Dlg1 can pull down Cx43 from HaCaT cell extracts, confirming that the two proteins can form a complex in keratinocytes. Taken together, our data indicate that Dlg1 is a binding partner of Cx43 in keratinocytes.

### Dlg1 controls Cx43 plasma membrane location in normal keratinocytes

**Fig. 3.**
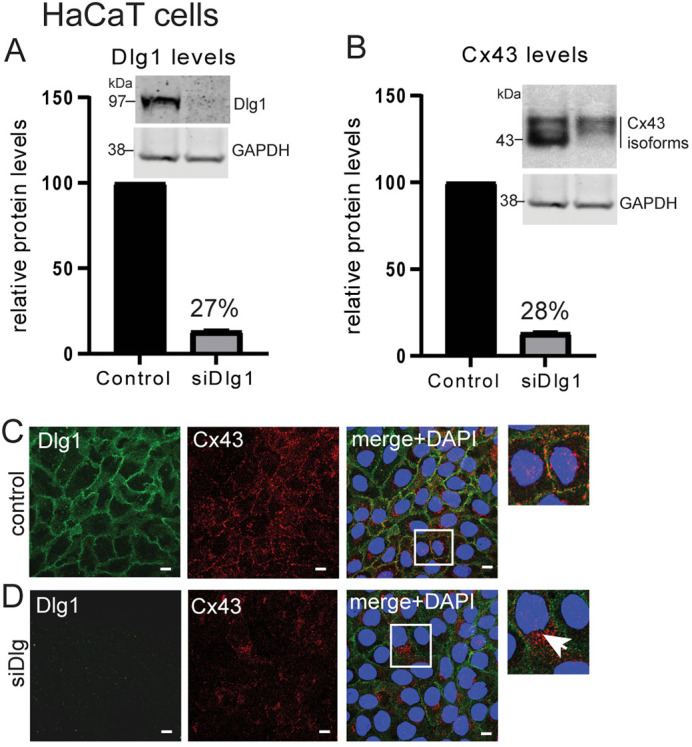
**Dlg1 depletion leads to reduced Cx43 levels and reduced appearance on the plasma membrane.** (A) Graph of Dlg1 levels relative to GAPDH in HaCaT cells treated with a control siRNA or treated with an siRNA against Dlg1 (used at 40 nM). (B) Graph of Cx43 levels relative to GAPDH in HaCaT cells treated with a control siRNA or treated with an siRNA against Dlg1. Representative western blots are shown above each graph. The same GAPDH blot is shown as a loading control in each case because the data come from one western blot experiment (see Fig. S2). The data are the mean±s.d. from three separate experiments. (C,D) Dlg1 (green) and Cx43 (red) antibody staining of HaCaT cells treated with (C) a control siRNA (control) or (D) siRNA against Dlg1 (siDlg). In the merge panel, the brightness in the green Dlg1 channel has been enhanced to show location of residual Dlg1. The white arrow in the boxed image in D indicates Cx43 intracytoplasmic staining. All images representative of at least three repeats. Scale bars: 10 µm.

Next, we investigated whether Dlg1 has a regulatory interaction with Cx43. HaCaT cells were treated with an siRNA pool to deplete Dlg1 (siDlg). Western blotting quantification showed that siRNA treatment significantly reduced Dlg1 levels to 27% of control siRNA-treated HaCaT cells (Fig. 3A). When levels of Dlg1 were reduced, Cx43 protein levels were also reduced (to 28%) compared to control siRNA-treated cells (Fig. 3B). Confocal microscopy revealed that knockdown of Dlg1 by siRNA resulted in overall loss of plasma membrane-associated Cx43 (compare Fig. 3C and D). The remaining Cx43 was mostly found in the cytoplasm in perinuclear regions (Fig. 3D, boxed image, arrow). Residual Dlg1 remained at the plasma membrane (Fig. 3D, enlarged image)

**Fig. 4.**
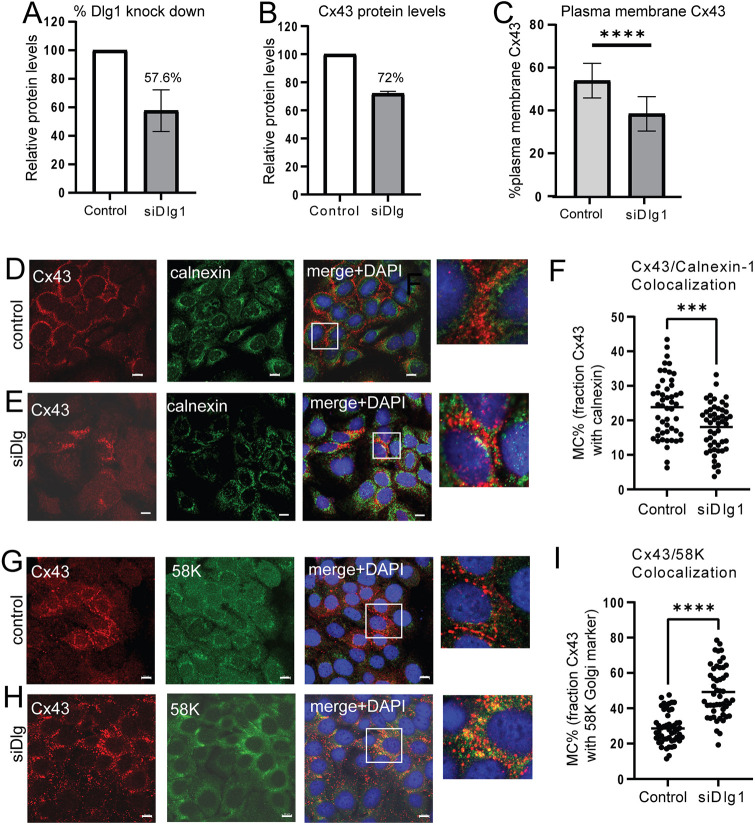
**Dlg1 controls trafficking of Cx43 from the Golgi.** (A) Graph of Dlg1 levels relative to GAPDH in HaCaT cells treated with a control siRNA or treated with an siRNA against Dlg1 (used at 20 nM). Half the concentration of siDlg1 was used in this experiment compared to the experiment in Fig. 3 to allow better visualisation of Cx43. (B) Graph of Cx43 levels relative to GAPDH in HaCaT cells treated with a control siRNA or treated with an siRNA against Dlg1. Graphs show the mean±s.d. from three separate experiments. (C) Quantification of Cx43 levels on the plasma membrane of 50 cells treated with control or Dlg1 siRNA. ****P<0.0001 (two-tailed *t*-test). (D,E) Cx43 (red) and calnexin (an ER-resident protein; green) antibody staining of HaCaT cells treated with (D) a control siRNA or (E) siRNA against Dlg1 (siDlg). (F) Manders’ colocalisation coefficient (MC) measurement of co-occurrence of Cx43 and ER staining. The mean is marked. ***P<0.0004 (two-tailed *t*-test). 50 individual cells were analysed for each treatment group. (G,H) Cx43 (red) antibody and 58K staining (green) of HaCaT cells treated with (G) control siRNA or (H) with siRNA against Dlg1 (siDlg). (I) Manders’ colocalisation coefficient (MC) measurement of co-occurrence of Cx43 and Golgi staining. The mean is marked. ****P<0.0001 (two-tailed *t*-test). 50 individual cells were analysed for each treatment group. In D, E, G and H, Merge+DAPI, merged imaged of red and green channels plus DAPI staining in blue to visualise nuclei. Enlarged sections of each merged image (white boxed areas) are shown on the right-hand side. All images representative of at least three repeats. Scale bars: 10 µm.

To investigate the cytoplasmic location of Cx43 upon Dlg1 depletion we co-stained cells with antibodies against Cx43, and calnexin, as an ER-resident protein, or with Golgi marker 58K or Golgi Tracker (Fig. S1), to locate the Golgi. For this experiment, the amount of Dlg1 siRNA pool transfected into the cells was halved to visualise Cx43 staining more easily and because it is known that Dlg1 depletion flattens cells shape, which might have a knock-on effect on Cx43 location (Rivera et al., 2009). Using this strategy, Dlg1 levels were reduced by 42.4% (Fig. 4A) and Cx43 levels were reduced by 28% (Fig. 4B) resulting in a 29% decrease of Cx43 on the plasma membrane (Fig. 4C). First, we investigated the presence of Cx43 in the ER (Fig. 4D,E, boxed areas). Changes in colocalisation of Cx43 and calnexin upon Dlg1 depletion were measured by assessing the Manders’ colocalisation coefficient. Using this approach allowed selection of thresholds and colocalisation measurements to be as independent of signal intensity as possible, while allowing an appropriate background signal level to be set. Only a limited overlap of calnexin and Cx43 antibody staining in control HaCaT cells was observed and there was a statistically significant reduction (*P*<0.05) in Cx43 colocation with the ER marker in cells treated with siRNA against Dlg1 (Fig. 4F). Next, we examined possible localisation of Cx43 in the Golgi. Cx43 showed significant colocalisation with 58K in HaCaT cells treated with Dlg1 siRNA (Fig. 4H) compared to cells treated with a control siRNA (Fig. 4G). An increase in Manders' colocalisation coefficient from 28.6% to 49.2% (*P*<0.0001) was observed comparing Cx43 and 58K colocation in mock-transfected cells with that in siRNA Dlg1-treated cells (Fig. 4I). A very similar result was obtained using Golgi Tracker as a Golgi marker (Fig. S1). Golgi staining was somewhat diffuse in these experiments. The HaCaT cells were grown under conditions where some differentiation can occur and keratinocyte differentiation has been shown to cause changes in the Golgi, resulting in diffuse staining (Mahanty et al., 2019). However, these changes do not affect our conclusion that the data suggest that loss of Dlg1 caused relocation of Cx43 to a cytoplasmic Golgi compartment.

**Fig. 5.**
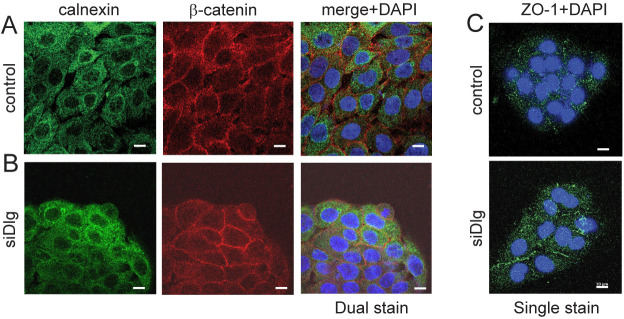
**Depletion of Dlg1 does not change calnexin location in the ER/Golgi, and plasma membrane location of β-catenin and ZO-1 is unaltered.** (A,B) Calnexin (green) and β-catenin (red) antibody staining of HaCaT cells treated with a (A) control siRNA (control) or (B) siRNA against Dlg1 (siDlg) used at 40 nM. Merge+DAPI, merged images of red and green channels plus DAPI staining in blue to visualise nuclei. (C) ZO-1 (green) antibody staining of HaCaT cells treated with a control siRNA (control) or siRNA against Dlg1 (siDlg). Nuclei are stained with DAPI. All images representative of at least three repeats. Scale bars: 10 µm.

Dlg1 is a so-called scaffolding protein known to control cell polarity (James and Roberts, 2016), thus changes in Cx43 location upon depletion of Dlg1 could be due to changes in cell shape, which might lead to remodelling of the plasma membrane. Comparison of calnexin location in the presence and depletion of Dlg1 showed little change to this intracellular compartment. Moreover, staining for β-catenin showed no gross disruption to the plasma membrane (Fig. 5, compare A and B). Finally, the localisation of the known Cx43-interacting protein ZO-1, a PDZ protein highly related to Dlg1, to the plasma membrane was found to be unaltered (Fig. 5C), suggesting that plasma membrane integrity was maintained.

### Dlg1 does not control Cx43 mRNA expression but alters Cx43 protein levels

**Fig. 6.**
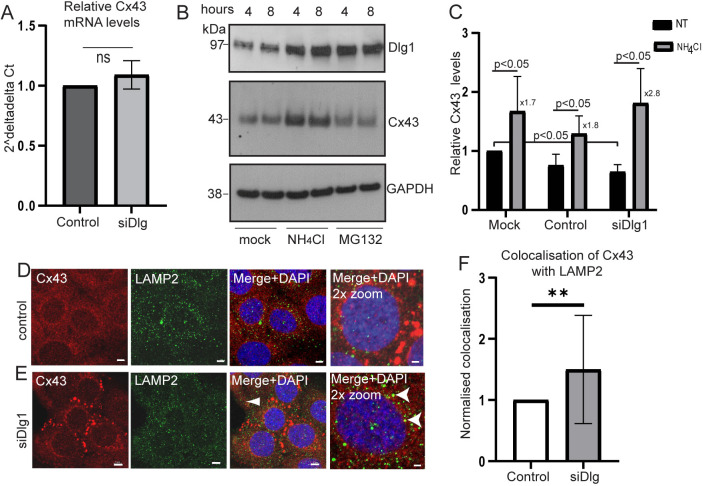
**Dlg1 depletion does not alter Cx43 mRNA expression but increases Cx43 protein degradation.** (A) qRT-PCR analysis of Cx43 mRNA levels in control siRNA-transfected (control) or Dlg1 siRNA-transfected (siDlg) HaCaT cells. Data are expressed as changes in Cx43 mRNA levels relative to changes in GAPDH mRNA levels (2^−ΔΔCt^). The mean±s.d. from three separate experiments are shown. ns, not significant (two-tailed *t*-test). (B) HaCaT cells were either mock-treated or treated with 10 mM NH_4_Cl or 10 µM MG132 for either 4 or 8 h. Cell lysates were harvested, and western blots performed to detect Dlg1, Cx43 and GAPDH, as a loading control. (C) Graph of quantification of protein levels of Cx43 in mock-transfected cells (mock), control siRNA-transfected cells (control) or in Dlg1 siRNA-transfected cells (siDlg) comparing levels in untreated (NT) and NH_4_Cl-treated cells. Data are expressed relative to GAPDH expression. Levels of Cx43 in cells not treated with NH_4_Cl are set at 1 to allow comparison of changes in Cx43 levels between control and Dlg1 siRNA-transfected cells. The fold change in Cx43 levels between NT and NH_4_Cl groups is shown to the side of the three NH_4_Cl bars. The mean±s.d. from three separate western blot experiments is shown. *P*<0.05 where indicated (two-tailed Mann–Whitney U-test). (D,E) Confocal immunofluorescence microscopy images of HaCaT cells treated with (D) a control siRNA or (E) with an siRNA against Dlg1 and stained with Cx43 and LAMP2 to detect the lysosomes. Cx43, red staining; Dlg1, green staining. Nuclei are stained with DAPI. Arrowheads indicate limited Cx43 and Dlg1 colocalisation. All images representative of at least three repeats. Scale bars: 10 µm (main image in D and E); 5 µm (2× zoom images in D and E). (F) Graph showing the mean±s.d. of the Manders' colocalisation coefficient quantification of Cx43 colocalisation with LAMP2; 50 cells were analysed in each of the control siRNA and Dlg1 siRNA (20 µM siRNA treatment, see Fig. 4) treatment groups. **P<0.01 (two-tailed *t*-test).

Dlg1 depletion resulted in reduced Cx43 protein levels (Fig. 3B), so we determined whether Dlg1 controlled Cx43 mRNA expression by quantitative reverse transcriptase PCR (qRT-PCR). Knockdown of Dlg1 did not alter Cx43 mRNA levels significantly (Fig. 6A). Western blot analysis of Cx43 protein levels in HaCaT cell lysates was used to determine the contribution of the lysosomes versus the proteasome in Cx43 degradation. Treatment of HaCaT cells with NH_4_Cl to inhibit the lysosomes resulted in an increase in Cx43 levels indicating that Cx43 can be subject to lysosomal degradation in these cells. In contrast, MG132 treatment to inhibit the proteasome did not lead to altered Cx43 levels (Fig. 6B). MG132 treatment was successful because increased levels of Dlg1, a protein known to be targeted for proteasomal degradation (James and Roberts, 2016), was observed (Fig. 6B). Next, we examined whether lysosomal inhibition by NH_4_Cl treatment altered Cx43 levels in the presence or depletion of Dlg1. Cx43 levels were inherently lower in Dlg1 siRNA-transfected cells (as seen in Fig. 3B) compared to mock-treated (Mock NT) and control siRNA-treated (Cntrl NT) cells (Fig. 6C). However, lysosomal inhibition resulted in a statistically significant increase in Cx43 levels in cells transfected with Dlg1 siRNA (Fig. 6C). We stained mock transfected and Dlg1 siRNA-treated HaCaT cells with antibodies against Cx43 and LAMP2 to detect the lysosomes. Like the Golgi, lysosome staining becomes more diffuse in keratinocytes which are differentiating (Mahanty et al., 2019), and this is clearly the case in Fig. 6D,E. The protocol for siRNA depletion of Dlg1 was the same as for Fig. 4 giving a 28% knockdown of Cx43. As previously observed, siRNA Dlg1 knockdown resulted in loss of plasma membrane Cx43 and relocation to a perinuclear location but very little colocalisation with LAMP2 was observed (Fig. 6E, merge+DAPI 2× zoom, white arrowheads). Quantification of Cx43 colocalisation with LAMP2 as determined from the Manders’ colocalisation coefficient showed that only 1% of cellular Cx43 was located in the lysosomes (Fig. 6F). However, the amount of colocalisation increased by 50% upon Dlg1 depletion (Fig. 6F).

### Cx43 plasma membrane localisation and gap junctional communication is regulated by Dlg1

**Fig. 7.**
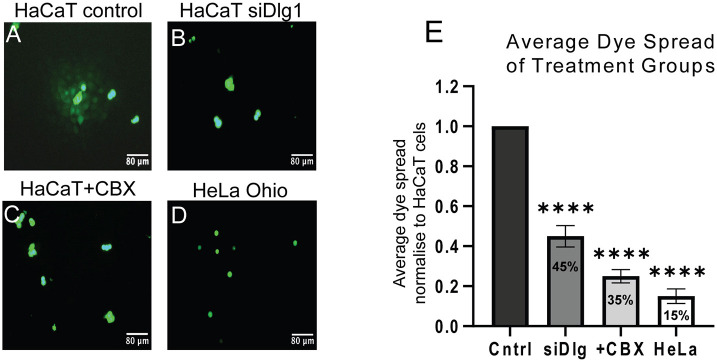
**Dlg1 depletion results in loss of gap junctional communication as measured by parachute assay in HaCaT cells.** Calcein dye transfer between donor cells [HaCaT (A–C) or HeLa Ohio cells (D)] and acceptor HaCaT cells. (A) Mock-treated HaCaT donor cells. (B) HaCaT donor cells treated with siRNA against Dlg1. (C) HaCaT donor cells treated with carbenoxolone (CBX) to block gap junctional communication. (D) HeLa Ohio donor cells that lack Cx43 expression parachuted onto HaCaT acceptor cells. (E) Quantification of the total number of acceptor cells receiving calcein dye from a directly adjacent donor cell per total number of donor cells, normalised to values from mock-treated HaCaT donor cells. At least 140 donor cells per treatment group were analysed. Percentage reduction in dye transfer is indicated on the bars on the graph. The data are the mean±s.d. from three separate experiments. ****P<0.0001 (Kruskal–Wallis test followed by Dunn's post-hoc test).

A reduction in Cx43 trafficking to the plasma membrane should result in loss of gap junctional communication. To examine this, we carried out parachute assays in HaCaT cells treated with siRNA against Dlg1 and compared these with mock-treated cells. In these experiments, we used the Dlg1 knockdown conditions shown in Fig. 3, which resulted in a reduction of 72% in Cx43 levels. Donor cells differed between treatment groups (HaCaT cells, HaCaT cells+siDlg1, HaCaT cells+CBX, HeLa Ohio cells), whereas acceptor HaCaT cells were kept consistent. In mock-treated HaCaT donor cells, dye (calcein) was efficiently transferred from the donor to acceptor HaCaT cells, showing that Cx43 forms functional gap junctions in these keratinocytes (Fig. 7A). However, in HaCaT donor cells with Dlg1 depletion, dye transfer was significantly reduced (Fig. 7B). HaCaT cells treated with carbenoxolone to block gap junctional communication and HeLa Ohio cells, which do not form Cx43 gap junctions (Elfgang et al., 1995), were used as negative controls for donor cells (Fig. 7C,D). Quantification of the average dye transfer revealed that Dlg1 knockdown inhibited dye spread significantly by 55% (Fig. 7E). Taken together, our data strongly suggest a role for Dlg1 in maintaining Cx43 GJs in the plasma membrane in keratinocytes.

## DISCUSSION

In this study, we addressed the hypothesis that Dlg1 is a protein binding partner of Cx43 and regulates Cx43 function in normal cells. We have demonstrated Cx43–Dlg1 colocalisation in cells of the epidermis: keratinocytes, dermal cells and adipocytes. We focused on keratinocytes given that we had previously shown Cx43 interaction with Dlg1 in precancerous HPV-infected cervical keratinocytes and cervical squamous cell cancer cell lines (MacDonald et al., 2012; Sun et al., 2015). Immunofluorescence microscopy, co-immunoprecipitation and GST pulldown together with analysis of colocalisation in tissues *in vivo* all revealed that Cx43 forms a complex with Dlg1. Our data show that a reduction in Dlg1 levels in keratinocytes leads to reduced levels of Cx43 protein in cells. Although Dlg1 can control gene expression (Gupta et al., 2018), Dlg1 depletion had no effect on Cx43 mRNA levels. This suggests that Dlg1 can regulate the Cx43 protein life cycle. Finally, loss of Dlg1 caused loss of functional plasma membrane Cx43 gap junction plaques, suggesting that Dlg1 is a physiologically relevant regulator of gap junction function.

Our previous studies have demonstrated that Cx43 and Dlg1 can interact in human papillomavirus type 16 (HPV16)-positive cervical tumour cells (MacDonald et al., 2012). The human papillomavirus E6 oncoprotein binds the central PDZ domain of Dlg1 and can target it for proteasomal degradation (Thomas et al., 2008), and we showed that a three-way complex of Cx43–Dlg1–E6 was present in cervical tumour cells (Sun et al., 2015). Our data showed that E6, when bound to the Cx43–Dlg1 complex, could inhibit accumulation of plasma membrane Cx43. Importantly, our new data reported here demonstrate that HPV E6 is not necessary for Cx43–Dlg1 interaction in normal cells and that Dlg1 itself is required for Cx43 function in gap junctional communication. An intriguing possibility is that the Cx43–Dlg1 pathway is hijacked by viral oncoproteins to result in a reduction in plasma membrane Cx43 and loss of intercellular communication, a tumour-promoting pathway.

In mammalian cells, Dlg1 colocates with adherens junction proteins and is required for adherence junction assembly and maintenance by forming a ternary complex with Scribble and a Rho guanine nucleotide exchange factor, SGEF, to regulate formation and maintenance of adherens junctions (Awadia et al., 2019; Bonello et al., 2019; Laprise et al., 2004). Adherens junctions are critical for delivery of connexons to the plasma membrane (Meyer et al., 1992). Thus, a functional interaction between Dlg1 and gap junction formation is likely. Dlg1 is found on the cytoplasmic side of the plasma membrane and is known to interact indirectly with the actin cytoskeleton (Firestein and Rongo, 2001; Reuver and Garner, 1998) and microtubules (Asaba et al., 2003). Given that microtubules and F-actin are required for gap junction formation Dlg1 might regulate connexon delivery and stabilisation in the plasma membrane by linking gap junctions to other plasma membrane junctions and the cytoskeleton.

**Fig. 8.**
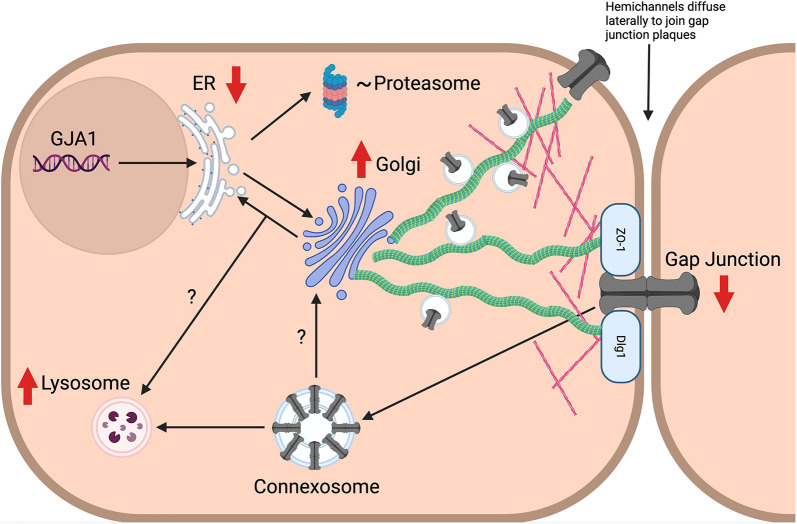
**Diagram of the Cx43 life cycle and consequences of Dlg1 depletion in a keratinocyte cell.** Dark brown indicates the plasma membrane. The light brown circle indicates the nucleus where the GJA1/double helix portion represents the gene encoding Cx43. Cellular organelles and plasma membrane junctions are labelled. Hemichannels and gap junctions are indicated in grey. Microtubules are shown in green. Actin filaments are shown in pink. Light blue lozenges indicate the PDZ proteins ZO-1 and Dlg1. Black arrows indicate the normal Cx43 life cycle. Red arrows indicate the observed changes (up or down arrows indicate increased or decreased levels respectively) to the Cx43 life cycle upon Dlg1 depletion. Lines with a question mark indicates possible ER/Golgi-related routes to Cx43 degradation when Dlg1 levels are reduced. Created with Biorender.com.

We found that there was a 29% reduction in plasma membrane Cx43 even at low levels of Dlg1 depletion (42.4% Dlg reduction in Fig. 4). Concomitant with this Cx43 plasma membrane reduction, there was an almost two-fold increase in levels of Cx43 in the Golgi compartment, suggesting that Cx43 became relocated to this compartment due to a reduction in Dlg1. As a ‘scaffolding protein’ Dlg1 can act as a docking and organisation nexus for partner proteins (Bonello et al., 2019) and can transport and recruit vesicular trafficking proteins to the plasma membrane (Fourie et al., 2014; Musa et al., 2020; Saraceno et al., 2014; Underhill et al., 2015) suggesting that Dlg1 could be involved in trafficking of Cx43 to the plasma membrane. However, this is unlikely given that we found no evidence of Dlg1 colocalisation with Cx43 at intracellular sites (Fig. 3). The Cx43-positive Golgi staining might include autophagosomes and/or lysosomes accumulating near the Golgi suggesting back tracking from the plasma membrane and accumulation in the Golgi followed by autophagosomal/lysosomal degradation. This is the route taken for degradation of E-cadherin upon depletion of Scribble (Lohia et al., 2012). Fig. 8 shows a summary diagram of changes to the Cx43 life cycle due to depletion of Dlg1.

ZO-1 is a well-recognised regulator of Cx43 gap junction formation and size (Ambrosi et al., 2016; Gourdie et al., 2006; Hunter et al., 2005; Rhett et al., 2010; Sorgen et al., 2004; Zheng et al., 2019). Although ZO-1 and Dlg1 are related proteins, Dlg1 does not bind the terminal five amino acids of Cx43 (MacDonald et al., 2012) indicating that each protein might have a different mode of interaction with Cx43 and might not complete for Cx43 binding. We hypothesise that these two MAGUK proteins could synergistically facilitate and/or stabilise Cx43 formation into gap junctions on the plasma membrane. Previously, another connexin, Cx32 has been shown to bind to Dlg1 (Singh and Lampe, 2003). Further studies indicated that Cx32 interacts with the GUK domain of Dlg1 (Stauch et al., 2012), which led to changes in levels and cellular localisation of Dlg1 (Duffy et al., 2007). Our previous studies have indicated that the GUK domain of Dlg1 is involved in Cx43 interaction (MacDonald et al., 2012). Future studies will examine the molecular determinants of the Cx43–Dlg1 complex formation. Understanding the precise molecular details of the interaction might lead to novel therapies to improve wound healing such as those designed to inhibit the ZO-1–Cx43 interaction (Montgomery et al., 2018, 2021)

## MATERIALS AND METHODS

### Human tissues

Formalin-fixed human abdominal skin tissue from three patients, taken during replacement of breast tissue following mastectomy, were obtained with ethical permission (NHS GG&C Biorepository). Informed consent was obtained for all tissue donors, and all clinical investigation were conducted according to the principles expressed in the Declaration of Helsinki.

### Cell culture

NIKS cells (Flores et al., 1999) were grown in F-medium (Jeon et al., 1995) on mitomycin C-treated 3T3 fibroblast feeder layer cells at a seeding ratio of 1:5 fibroblasts to keratinocytes (fibroblasts 1×10^6^; keratinocytes 2×10^5^ cells/100 mm dish). Fibroblasts were removed by trypsinisation prior to protein purification or growth on coverslips. HaCaT cells (Boukamp et al., 1988), HeLa Ohio cells and HEK293 cells were grown in DMEM with 10% fetal calf serum (Invitrogen, Paisley, UK). All cells were maintained in a humidified incubator with 5% CO_2_ at 37°C.

### Cell treatments

Knockdown of Dlg1 in HaCaT cells was achieved by transfection with a Dharmacon ON-TargetplusSMARTpool against Dlg1 in RNAiMax transfection reagent (Invitrogen, Paisley UK) at 40 nM (Figs 3, 5, 7) or 20 nM (Figs 4, 6). siGLO (Thermo Fisher Scientific) was used as a non-target siRNA control and to monitor transfection efficiency. Endosomal/lysosomal inhibition was carried out by treatment of cells with either 10 mm NH_4_Cl (Sigma, Poole, UK) or 200 µM chloroquine (Sigma) for 8 h at 37°C (MacDonald et al., 2012). Proteasome inhibition was carried out using MG132 (Sigma) at 10 µM for 4 or 8 h (MacDonald et al., 2012).

### qRT-PCR and analysis

Dlg1 depletion in HaCaT cells was carried out as above. RNA was prepared from siRNA-transfected cells using Qiagen RNeasy extraction (Qiagen, Manchester, UK) exactly as described in the manufacturer's protocol. cDNA was synthesised using a Maxima cDNA synthesis kit (Thermo Fisher Scientific, UK) with DNase digestion according to the manufacturer's protocol. The Cx43 forward primer was 5′-CTGGGTCCTGCAGATCATATTT-3′. The Cx43 reverse primer was 5′-GGCAACCTTGAGTTCTTCCT-3′. The Cx43 probe was 5′-CCCACACTCTTGTACCTGGCTCAT-3′. The GAPDH forward primer was 5′-GAAGGTGAAGGTCGGAGT-3′. The GAPDH reverse primer was 5′-GAAGATGGTGATGGGATTTC-3′. The GAPDH probe was 5′-CAAGCTTCCCGTTCTCAGCC-3′ (Eurofins, Livingston, UK). The final concentrations of primer and probe in each reaction were 900 nM and 100 nM respectively. qRT-PCR was carried out using a 7500 Real Time PCR System (Thermo Fisher Scientific) with the following conditions: 1 cycle of 50°C for 2 min, 1 cycle of 95°C for 3 min, then 40 cycles of 95°C for 10 s followed by 60°C for 1 min. Each sample was run in triplicate. ΔΔCt values were calculated from results which were then used to generate an average expression ratio of Cx43 cDNA in cells depleted of Dlg1 compared to mock-treated cells.

### Protein extract preparation and western blotting

Cells were washed twice in PBS at 4°C and lysed in 2× BOLT protein loading buffer (Invitrogen). Protein extracts were syringe-passaged through a 22-gauge needle 15 times then sonicated in a Sonibath (Kerry Ultrasonics, Hitchin, UK) for 3×30 s pulses. The samples were boiled at 100°C for 5 min before loading on a 12% NuPAGE gel (Invitrogen) and electrophoresed at 150 V for 1 h in 1× MES buffer. Proteins were transferred to a nitrocellulose membrane using the iBlot transfer kit and iBlot Gel Transfer Stacks (Invitrogen) as per the manufacturer's instructions. Membranes were blocked in 5% (w/v) milk powder in phosphate-buffered saline (PBS) with 0.1% Tween 20 (PBST) at room temperature for 1 h. Membranes were washed three times in PBST [or Tris-buffered saline (TBS) with 0.1% Tween 20 (TBST) for phosphoproteins] for 5 min each then incubated with primary antibody. A polyclonal antibody C-6219 against Cx43 (cat. no. C6219, Sigma, Poole, UK) was used in western blotting at 1:5000. Dlg1 monoclonal antibody 2D11 (cat. no. sc9961, Santa Cruz Biotechnology, CA, USA) and the GAPDH antibody clone 6C5 (cat. no. H86504M, Biodesign) were used at 1:1000 dilution. The blots were incubated in their respective antibody for 1 h at room temperature or overnight at 4°C. The blots were washed three times in PBST or TBST for 5 min. They were then placed in secondary antibody for 1 h [HRP-linked goat anti-mouse-IgG or goat anti-rabbit-IgG (Pierce, Thermo Fisher Scientific)] were used at a 1:2000 dilution. Blots were washed 3 times in PBST for 5 min before incubation with ECL western blot substrate. The blots were exposed to X-ray film (Thermo Fisher Scientific) and processed in an X-Omat processor or imaged using a LI-COR Odyssey imager. Signal intensity for Cx43 and Dlg1 bands was quantified and normalised against the signal for GAPDH. All experiments were performed as minimum of three biological replicates. Fig. S2 shows larger portions of all images for blot transparency.

### Confocal immunofluorescence microscopy and quantification

Cells were grown on sterile coverslips until 90% confluent, then washed three times with PBS. For Cx43 and LAMP-2 colocalisation, cells were fixed with 4% PFA for 15 min at room temperature. For all other experiments, cells were fixed with 100% ice-cold methanol for 10 min at 4°C. Cells were permeabilised in acetone for 1 min followed by three 5 min washes in PBS and incubation at room temperature for 1 h with 10% (v/v) donkey serum to block. Primary antibodies were diluted using 5% (v/v) donkey serum and incubated for either 1 h at room temperature or overnight at 4°C. Coverslips were washed five times with PBS. Antibodies were a polyclonal raised in rabbits against a synthetic peptide corresponding to residues 363–382 of Cx43 (1:500; kindly provided by Edward Leithe, Oslo University Hospital, Norway), and Dlg1 mouse monoclonal antibody (1:250; cat. no. sc-9961, Santa Cruz Biotechnology). ZO-1 mouse antibody (cat. no. 610966, Thermo Fisher Scientific) was used at 1:500. 58K mouse monoclonal antibody (cat. no. ab27043, Abcam) was used at 1:100 and Golgi Staining Kit Cytopainter (Golgi Tracker; cat. no. ab139483, Abcam) was used to stain the Golgi apparatus according to the manufacturer's instructions. Calnexin-1 polyclonal rabbit antibody (cat. no. ADI-SPA-860, Enzo Life Sciences) was used at 1:100 to stain the ER. LAMP-2 monoclonal mouse antibody 66301-1-Ig (Proteintech) was used at 1:100 to stain the lysosomes. Anti-β-catenin antibody was used at 1:200 (cat. no. 610154, BD Transduction Laboratories). EEA1 mouse monoclonal antibody (cat. no. ab15846, Abcam) was used at 1:200 to stain early endosomes. Alexa Fluor secondary antibodies were diluted 1:500 in blocking solution and added to the cells for 1 h, protected from light, prior to 5 washes in PBS, followed by one wash in distilled (d)H_2_O. Coverslips were mounted on glass slides with ProLong™ Gold Antifade Mountant with DAPI (Invitrogen).

For staining of paraffin-embedded human skin tissue, sections on slides were de-paraffined, and antigen retrieval was performed using sodium citrate (10 mM, pH 6.0). Sections were then washed gently in PBS before blocking for 1 h as above. Incubation with Cx43 (1:500) and Dlg1 (1:50) primary antibodies was carried out overnight at 4°C. Subsequent steps were performed as above. Control samples including secondary antibodies with no primary antibodies were carried out for all experiments, with an additional control of 1 µg/ml rabbit IgG in place of primary antibody being included for human skin tissue staining. Samples were examined using a Zeiss LSM 710 confocal microscope and Zen black software (Zeiss) was used for capturing images. Data shown are representative of at least three different experiments.

Images were analysed for quantification of colocalisation using Zen blue software (Zeiss). As Cx43 signal levels were expected to vary between untreated and siRNA-treated cells, threshold setting and colocalisation measurements were selected to be as independent of signal intensity as possible, while allowing an appropriate background signal level to be set. Thresholds for colocalisation were set using single stains of Cx43, calnexin-1, LAMP-2, Golgi Tracker, 58K and EEA1 imaged in both red and green channels under the same conditions as in the test samples. Cells were outlined using the Zeiss software drawing tool, and Manders’ colocalisation coefficient (M_1_) was measured for each outlined cell (Manders et al., 1993); 50 individual cells were analysed for each treatment group.

Quantification of the proportion of total Cx43 on the plasma membrane was performed using ImageJ/Fiji^®^ software. Using the free-hand drawing tool, two outlines were created for each cell, one surrounding the entire cell and the other covering the cell minus the plasma membrane (as indicated by β-catenin plasma membrane staining). The proportions of Cx43 staining were analysed using integrated density values obtained from each of these outlines. To account for background signal, three areas with no Cx43 staining were selected in each image and the average value of these areas were subtracted from the raw values. Cx43 signal level on the plasma membrane was calculated by subtracting the value of the outline not containing the plasma membrane from the outline containing the plasma membrane. The resulting value was then divided by the value of the outline covering the entire cell to give the portion of total cellular Cx43 on the plasma membrane; 50 individual cells were analysed for each treatment group.

### Co-immunoprecipitation

Co-immunoprecipitation was carried out as previously described (MacDonald et al., 2012). Briefly, cells were lysed in RIPA buffer [20 mM Tris-HCl pH 7.5, 150 mM NaCl, 1 mM Na_2_EDTA, 1 mM EGTA, 1% (v/v) NP-40 1% (w/v) sodium deoxycholate, 2.5 mM sodium pyrophosphate, 1 mM β-glycerophosphate, 1 mM Na_3_VO_4_, 1 µg/ml leupeptin] containing either 0.5% (v/v) NP-40 (Nonidet-40) or Triton X-100. Primary antibodies used were control rabbit IgG (Sigma, Poole, UK), Cx43 C-6219 (Sigma) and anti-Dlg1 polyclonal antibody H-60 (Santa Cruz Biotechnology). Samples were boiled at 100°C for 5 min before proteins were resolved by SDS-PAGE.

### GST pull-down

GST–Dlg1 preparation and purification was carried out as described (Gardiol et al., 1999). 5 μg of bacterial lysate expressing GST only or GST–Dlg was incubated with 20 μl of pre-cleared glutathione–Sepharose 4B beads (GE Healthcare, ref. no. 17075601) at 4°C for 1 h on a rotating shaker. Samples were made up to 100 μl with RIPA lysis buffer (see above). Then the beads were washed twice with 500 μl RIPA lysis buffer and pelleted by centrifugation at 4°C at 8600 ***g*** for 30 s. The beads were incubated with 100 μg HaCaT cell extracts at 4°C overnight with rotation. Following this, beads were washed three times with RIPA lysis buffer then resuspended in 10 µl protein loading buffer. Samples were analysed by SDS-PAGE and western blotting.

### Parachute assay

Donor cells (three plates of HaCaT cells, one plate of HeLa Ohio cells) were grown in 60 mm plates until they reached 30–50% confluence, at which point one plate of HaCaT cells was treated with siRNA targeting Dlg1. Cells were incubated for a further 24 h before being treated with 2.5 µM calcein-AM (C1300MP, Thermo Fisher Scientific) diluted in PBS plus 1 mM Ca^2+^ at 37°C for 30 min with gentle rocking every 5 min. Calcein-AM is a membrane permeable compound which is converted intracellularly into green fluorescent calcein, which can pass through gap junctions. Cells were washed three times in PBS plus 1 mM Ca^2+^ and incubated at 37°C for 30 min in fresh medium. Following this, donor cells were washed twice and incubated with 1 µM CellTracker™ CM-DiI (C7000, Thermo Fisher Scientific) for 5 min at 37°C after which they were wrapped in tinfoil to protect them from light and incubated for a further 15 min at 4°C. CM-Dil is a red fluorescent dye that can pass through plasma membranes but becomes trapped within the cell, therefore allowing distinction of red and green ‘donor’ cells from green ‘acceptor’ cells. Cells were washed three times and trypsinised before being pelleted by centrifugation at 283 ***g*** for 5 min. Cells were resuspended and pelleted as before and then resuspended and counted. 50 µM carbenoxolone (CBX; C4790-1G, Sigma) was added to HaCaT cells and incubated for 30 min to block communication through gap junctions. Donor cells (HaCaT cells, HaCaT cells+siDlg1, HaCaT cells+CBX, HeLa Ohio Cells) were ‘parachuted’ onto 80% confluent ‘acceptor’ HaCaT cells, which were grown in a 12-well plate at a donor:acceptor cell ratio of 1:20. Each condition was run in triplicate. Cells were incubated at 37°C for 4 h 30 min to allow dye transfer to take place followed by imaging on an AMG EVOS imaging microscope. Gap junctional communication was assessed as the total number of acceptor cells receiving calcein green dye from a directly adjacent donor cell per total number of donor cells. At least 140 donor cells per treatment group were analysed.

### Statistical analysis

An unpaired two-tailed *t*-test was used to assess significance in the colocalisation analyses. For western blots, Dlg1 and Cx43 values were normalised to GAPDH for each sample. Significance was assessed using an unpaired two-tailed *t*-test or a two-tailed Mann–Whitney *U*-test in the case of the lysosomal inhibition experiment. For qRT-PCR, an unpaired two-tailed *t*-test was performed on 2^−ΔΔCt^ values to assess significance. For the parachute assay, results were expressed as the average number of acceptor cells receiving dye from a directly adjacent donor cell per donor cell. Significance was assessed using a Kruskal–Wallis test followed by Dunn's post hoc test to determine differences between the individual treatment groups. Data shown are the result of at least three biological replicates.

## Supplementary Material

10.1242/joces.259984_sup1 Supplementary information

## References

[JCS259984C1] Aasen, T., Johnstone, S., Vidal-Brime, L., Lynn, K. S. and Koval, M. (2018). Connexins: synthesis, post-translational modifications, and trafficking in health and disease. *Int. J. Mol. Sci.* 19, 1296. 10.3390/ijms19051296 29701678 PMC5983588

[JCS259984C2] Aasen, T., Leithe, E., Graham, S. V., Kameritsch, P., Mayán, M. D., Mesnil, M., Pogoda, K. and Tabernero, A. (2019). Connexins in cancer: bridging the gap to the clinic. *Oncogene* 38, 4429-4451. 10.1038/s41388-019-0741-6 30814684 PMC6555763

[JCS259984C3] Allen-Hoffmann, B. L., Schlosser, S. J., Ivarie, C. A. R., Meisner, L. F., O'Connor, S. L. and Sattler, C. A. (2000). Normal growth and differentiation in a spontaneously immortalized near-diploid human keratinocyte cell line, NIKS. *J. Investig. Dermatol.* 114, 444-455. 10.1046/j.1523-1747.2000.00869.x 10692102

[JCS259984C4] Ambrosi, C., Ren, C., Spagnol, G., Cavin, G., Cone, A., Grintsevich, E. E., Sosinsky, G. E. and Sorgen, P. L. (2016). Connexin43 forms supramolecular complexes through non-overlapping binding sites for Drebrin, Tubulin, and ZO-1. *PLoS ONE* 11, e0157073. 10.1371/journal.pone.0157073 27280719 PMC4900556

[JCS259984C5] Asaba, N., Hanada, T., Takeuchi, A. and Chishti, A. H. (2003). Direct interaction with a kinesin-related motor mediates transport of mammalian discs large tumor suppressor homologue in epithelial cells*. *J. Biol. Chem.* 278, 8395-8400. 10.1074/jbc.M210362200 12496241

[JCS259984C6] Awadia, S., Huq, F., Arnold, T. R., Goicoechea, S. M., Sun, Y. J., Hou, T., Kreider-Letterman, G., Massimi, P., Banks, L., Fuentes, E. J. et al. (2019). SGEF forms a complex with Scribble and Dlg1 and regulates epithelial junctions and contractility. *J. Cell Biol.* 218, 2699-2725. 10.1083/jcb.201811114 31248911 PMC6683736

[JCS259984C7] Basu, R., Bose, A., Thomas, D. and Das Sarma, J. (2017). Microtubule-assisted altered trafficking of astrocytic gap junction protein connexin 43 is associated with depletion of connexin 47 during mouse hepatitis virus infection. *J. Biol. Chem.* 292, 14747-14763. 10.1074/jbc.M117.786491 28566289 PMC5592656

[JCS259984C8] Berthoud, V. M., Minogue, P. J., Laing, J. G. and Beyer, E. C. (2004). Pathways for degradation of connexins and gap junctions. *Cardiovasc. Res* 62, 256-267. 10.1016/j.cardiores.2003.12.021 15094346

[JCS259984C9] Bonello, T. T., Choi, W. and Peifer, M. (2019). Scribble and Discs-large direct initial assembly and positioning of adherens junctions during the establishment of apical-basal polarity. *Development* 146, dev180976. 10.1242/dev.180976 31628110 PMC6899021

[JCS259984C10] Bonilha, V. L. and Rodriguez-Boulan, E. (2001). Polarity and developmental regulation of two PDZ proteins in the retinal pigment epithelium. *Invest. Ophthalmol. Vis. Sci.* 42, 3274-3282. 11726633

[JCS259984C11] Boukamp, P., Petrussevska, R. T., Breitkreutz, D., Hornung, J., Markham, A. and Fusenig, N. E. (1988). Normal keratinization in a spontaneously immortalized aneuploid human keratinocyte cell line. *J. Cell Biol* 106, 761-771. 10.1083/jcb.106.3.761 2450098 PMC2115116

[JCS259984C12] Butkevich, E., Hülsmann, S., Wenzel, D., Shirao, T., Duden, R. and Majoul, I. (2004). Drebrin is a novel connexin-43 binding partner that links gap junctions to the submembrane cytoskeleton. *Curr. Biol.* 14, 650-658. 10.1016/j.cub.2004.03.063 15084279

[JCS259984C13] Chanson, M., Watanabe, M., O'Shaughnessy, E. M., Zoso, A. and Martin, P. E. (2018). Connexin communication compartments and wound repair in epithelial tissue. *Int. J. Mol. Sci.* 19, 1354. 10.3390/ijms19051354 29751558 PMC5983803

[JCS259984C14] Duffy, H. S., Iacobas, I., Hotchkiss, K., Hirst-Jensen, B. J., Bosco, A., Dandachi, N., Dermietzel, R., Sorgen, P. L. and Spray, D. C. (2007). The gap junction protein connexin32 interacts with the Src homology 3/hook domain of discs large homolog. *J. Biol. Chem.* 282, 9789-9796. 10.1074/jbc.M605261200 17284442

[JCS259984C90] Elfgang, C., Eckert, R., Lichtenberg-Frate, H., Butterweck, A., Traub, O., Klein, R. A., Hulser, D. F. and Willecke, K. (1995). Specific permeability and selective formation of gap junction channels in connexin-transfected HeLa cells. *J. Cell Biol.* 129, 806-817. 10.1083/jcb.129.3.805 PMC2120441 7537274

[JCS259984C15] Epifantseva, I. and Shaw, R. M. (2018). Intracellular trafficking pathways of Cx43 gap junction channels. *Biochim. Biophys. Acta Biomembr.* 1860, 40-47. 10.1016/j.bbamem.2017.05.018 28576298 PMC5731482

[JCS259984C16] Evans, W. H. (2015). Cell communication across gap junctions: a historical perspective and current developments. *Biochem. Soc. Trans.* 43, 450-459. 10.1042/BST20150056 26009190

[JCS259984C17] Falk, M. M., Kells, R. M. and Berthoud, V. M. (2014). Degradation of connexins and gap junctions. *FEBS Lett.* 588, 1221-1229. 10.1016/j.febslet.2014.01.031 24486527 PMC3989500

[JCS259984C18] Firestein, B. L. and Rongo, C. (2001). DLG-1 is a MAGUK similar to SAP97 and is required for adherens junction formation. *Mol. Biol. Cell* 12, 3465-3475. 10.1091/mbc.12.11.3465 11694581 PMC60268

[JCS259984C19] Flores, E. R., Allen-Hoffmann, B. L., Lee, D., Sattler, C. A. and Lambert, P. F. (1999). Establishment of the human papillomavirus type 16 (HPV-16) life cycle in an immortalized human foreskin keratinocyte cell line. *Virology* 262, 344-354. 10.1006/viro.1999.9868 10502513

[JCS259984C20] Fourie, C., Li, D. and Montgomery, J. M. (2014). The anchoring protein SAP97 influences the trafficking and localisation of multiple membrane channels. *Biochim. Biophys. Acta Biomembr.* 1838, 589-594. 10.1016/j.bbamem.2013.03.015 23535319

[JCS259984C21] Gardiol, D., Kühne, C., Glausinger, B., Lee, S. S., Javier, R. and Banks, L. (1999). Oncogenic human papillomavirus E6 proteins target the discs large tumour suppressor for proteasome-mediated degradation. *Oncogene* 18, 5487-5496. 10.1038/sj.onc.1202920 10523825

[JCS259984C22] Giepmans, B. N. G. and Moolenar, W. H. (1998). The gap junction protein connexin-43 interacts with the second PDZ domain of the zona occludens-1 protein. *Curr. Biol.* 8, 931-934. 10.1016/S0960-9822(07)00375-2 9707407

[JCS259984C23] Giepmans, B. N. G., Verlaan, I., Hengeveld, T., Janssen, H., Calafat, J., Falk, M. M. and Moolenaar, W. H. (2001). Gap junction protein connexin-43 interacts directly with microtubules. *Curr. Biol.* 11, 1364-1368. 10.1016/S0960-9822(01)00424-9 11553331

[JCS259984C24] Golub, O., Wee, B., Newman, R. A., Paterson, N. M. and Prehoda, K. E. (2017). Activation of Discs large by aPKC aligns the mitotic spindle to the polarity axis during asymmetric cell division. *eLife* 6, e32137. 10.7554/eLife.32137.018 29185419 PMC5706957

[JCS259984C25] Gourdie, R. G., Ghatnekar, G. S., O'quinn, M., Rhett, M. J., Barker, R. J., Zhu, C., Jourdan, J. and Hunter, A. W. (2006). The unstoppable connexin43 carboxyl-terminus: new roles in gap junction organization and wound healing. *Ann. N. Y. Acad. Sci* 1080, 49-62. 10.1196/annals.1380.005 17132774

[JCS259984C26] Gupta, P., Uner, O. E., Nayak, S., Grant, G. R. and Kalb, R. G. (2018). SAP97 regulates behavior and expression of schizophrenia risk enriched gene sets in mouse hippocampus. *PLoS ONE* 13, e0200477. 10.1371/journal.pone.0200477 29995933 PMC6040763

[JCS259984C27] Hunter, A. W., Barker, R. J., Zhu, C. and Gourdie, R. G. (2005). Zonal Occulens-1 alters connexin43 gap junction size and organization by influencing channel accretion. *Mol. Biol. Cell* 16, 5686-5698. 10.1091/mbc.e05-08-0737 16195341 PMC1289413

[JCS259984C28] James, C. D. and Roberts, S. (2016). Viral Interactions with PDZ domain-containing proteins—an oncogenic trait? *Pathogens* 5, 8. 10.3390/pathogens5010008 26797638 PMC4810129

[JCS259984C29] Jeon, S., Allen-Hoffman, B. L. and Lambert, P. F. (1995). Integration of human papillomavirus type 16 into the human genome correlates with a selective growth advantage of cells. *J. Virol.* 69, 2989-2997. 10.1128/jvi.69.5.2989-2997.1995 7707525 PMC188998

[JCS259984C30] Johnstone, S. R., Billaud, M., Lohman, A. W., Taddeo, E. P. and Isakson, B. E. (2012). Posttranslational modifications in connexins and pannexins. *J. Membr. Biol.* 245, 319-332. 10.1007/s00232-012-9453-3 22739962 PMC3954810

[JCS259984C31] Kang, E. Y., Ponzio, M., Gupta, P. P., Liu, F., Butensky, A. and Gutstein, D. E. (2009). Identification of binding partners for the cytoplasmic loop of connexin43: a novel interaction with β-tubulin. *Cell Commun. Adhes.* 15, 397-406. 10.1080/15419060902783833 19274588 PMC2889002

[JCS259984C32] Kim, S.-N., Kwon, H.-J., Im, S.-W., Son, Y.-H., Akindehin, S., Jung, Y.-S., Lee, S. J., Rhyu, I. J., Kim, I. Y., Seong, J.-K. et al. (2017). Connexin 43 is required for the maintenance of mitochondrial integrity in brown adipose tissue. *Sci. Rep.* 7, 7159. 10.1038/s41598-017-07658-y 28769076 PMC5540980

[JCS259984C33] Knoblich, J. A. (2008). Mechanisms of asymmetric stem cell division. *Cell* 132, 583-597. 10.1016/j.cell.2008.02.007 18295577

[JCS259984C34] Laird, D. W. (2006). Life cycle of connexins in health and disease. *Biochem. J* 394, 527-543. 10.1042/BJ20051922 16492141 PMC1383703

[JCS259984C35] Laprise, P., Viel, A. and Rivard, N. (2004). Human homolog of disc-large is required for adherens junction assembly and differentiation of human intestinal epithelial cells. *J. Biol. Chem.* 279, 10157-10166. 10.1074/jbc.M309843200 14699157

[JCS259984C36] Lauf, U., Giepmans, B. N. G., Lopez, P., Braconnot, S., Chen, S.-C. and Falk, M. M. (2002). Dynamic trafficking and delivery of connexons to the plasma membrane and accretion to gap junctions in living cells. *Proc. Natl. Acad. Sci. USA* 99, 10446-10451. 10.1073/pnas.162055899 12149451 PMC124935

[JCS259984C37] Leithe, E. and Rivedal, E. (2004). Ubiquitination and down-regulation of gap junction protein connexin-43 in response to 12-O-tetradecanoylphorbol 13-acetate treatment. *J. Biol. Chem.* 279, 50089-50096. 10.1074/jbc.M402006200 15371442

[JCS259984C38] Leithe, E., Mesnil, M. and Aasen, T. (2018). The connexin 43 C-terminus: a tail of many tales. *Biochim. Biophys. Acta Biomembr.* 1860, 48-64. 10.1016/j.bbamem.2017.05.008 28526583

[JCS259984C39] Lilly, E., Sellitto, C., Milstone, L. M. and White, T. W. (2016). Connexin channels in congenital skin disorders. *Semin. Cell Dev. Biol.* 50, 4-12. 10.1016/j.semcdb.2015.11.018 26775130 PMC4779425

[JCS259984C40] Lohia, M., Qin, Y. and Macara, I. G. (2012). The scribble polarity protein stabilizes E-cadherin/p120-catenin binding and blocks retrieval of E-cadherin to the golgi. *PLoS ONE* 7, e51130. 10.1371/journal.pone.0051130 23226478 PMC3511384

[JCS259984C41] Lorraine, C., Wright, C. S. and Martin, P. E. (2015). Connexin43 plays diverse roles in co-ordinating cell migration and wound closure events. *Biochem. Soc. Trans.* 43, 482-488. 10.1042/BST20150034 26009195

[JCS259984C42] MacDonald, A. I., Sun, P., Hernandez-Lopez, H., Aasen, T., Hodgins, M. B., Edward, M., Roberts, S., Massimi, P., Thomas, M., Banks, L. et al. (2012). A functional interaction between the MAGUK protein hDlg and the gap junction protein connexin 43 in cervical tumour cells. *Biochem. J.* 446, 9-21. 10.1042/BJ20111144 22657348

[JCS259984C43] Mahanty, S., Dakappa, S. S., Shariff, R., Patel, S., Swamy, M. M., Majumdar, A. and Setty, S. R. G. (2019). Keratinocyte differentiation promotes ER stress-dependent lysosome biogenesis. *Cell Death Dis.* 10, 269. 10.1038/s41419-019-1478-4 30890691 PMC6425001

[JCS259984C44] Manders, E. M. M., Verbeek, F. J. and Aten, J. A. (1993). Measurement of co-localization of objects in dual-colour confocal images. *J. Microsc.* 169, 375-382. 10.1111/j.1365-2818.1993.tb03313.x 33930978

[JCS259984C45] Marfatia, S. M., Byron, O., Campbell, G., Liu, S.-C. and Chishti, A. H. (2000). Human homologue of the Drosophila discs large tumor suppressor protein forms an oligomer in solution. Identification of the self-association site. *J. Biol. Chem.* 275, 13759-13770. 10.1074/jbc.275.18.13759 10788497

[JCS259984C46] Martin, P. E., Easton, J. A., Hodgins, M. B. and Wright, C. S. (2014). Connexins: sensors of epidermal integrity that are therapeutic targets. *FEBS Lett.* 588, 1304-1314. 10.1016/j.febslet.2014.02.048 24607543

[JCS259984C47] Meng, L. and Yan, D. (2020). NLR-1/CASPR anchors F-actin to promote gap junction formation. *Dev. Cell* 55, 574-587.e573. 10.1016/j.devcel.2020.10.020 33238150 PMC7725993

[JCS259984C48] Meyer, R. A., Laird, D. W., Revel, J. P. and Johnson, R. G. (1992). Inhibition of gap junction and adherens junction assembly by connexin and A-CAM antibodies. *J. Cell Biol.* 119, 179-189. 10.1083/jcb.119.1.179 1326565 PMC2289623

[JCS259984C49] Montgomery, J., Ghatnekar, G. S., Grek, C. L., Moyer, K. E. and Gourdie, R. G. (2018). Connexin 43-based therapeutics for dermal wound healing. *Int. J. Mol. Sci.* 19, 1778. 10.3390/ijms19061778 29914066 PMC6032231

[JCS259984C50] Montgomery, J., Richardson, W. J., Marsh, S., Rhett, J. M., Bustos, F., Degen, K., Ghatnekar, G. S., Grek, C. L., Jourdan, L. J., Holmes, J. W. et al. (2021). The connexin 43 carboxyl terminal mimetic peptide αCT1 prompts differentiation of a collagen scar matrix in humans resembling unwounded skin. *FASEB J.* 35, e21762. 10.1096/fj.202001881R 34246197 PMC8667734

[JCS259984C51] Müller, B. M., Kistner, U., Veh, R. W., Cases-Langhoff, C., Becker, B., Gundelfinger, E. D. and Garner, C. C. (1995). Molecular characterization and spatial distribution of SAP97, a novel presynaptic protein homologous to SAP90 and the Drosophila discs-large tumor suppressor protein. *J. Neurosci.* 15, 2354-2366. 10.1523/JNEUROSCI.15-03-02354.1995 7891172 PMC6578138

[JCS259984C52] Musa, H., Marcou, C. A., Herron, T. J., Makara, M. A., Tester, D. J., O'Connell, R. P., Rosinski, B., Guerrero-Serna, G., Milstein, M. L., Monteiro Da Rocha, A. et al. (2020). Abnormal myocardial expression of SAP97 is associated with arrhythmogenic risk. *Am. J. Physiol. Heart Circ. Physiol.* 318, H1357-h1370. 10.1152/ajpheart.00481.2019 32196358 PMC7311695

[JCS259984C53] Musil, L. S. and Goodenough, D. A. (1993). Multisubunit assembly of an integral plasma membrane channel protein, gap junction connexin43, occurs after exit from the ER. *Cell* 74, 1065-1077. 10.1016/0092-8674(93)90728-9 7691412

[JCS259984C54] Nakagawa, T., Futai, K., Lashuel, H. A., Lo, I., Okamoto, K., Walz, T., Hayashi, Y. and Sheng, M. (2004). Quaternary structure, protein dynamics, and synaptic function of SAP97 controlled by L27 domain interactions. *Neuron* 44, 453-467. 10.1016/j.neuron.2004.10.012 15504326

[JCS259984C55] O'Neill, A. K., Gallegos, L. L., Justilien, V., Garcia, E. L., Leitges, M., Fields, A. P., Hall, R. A. and Newton, A. C. (2011). Protein kinase Cα promotes cell migration through a PDZ-dependent interaction with its novel substrate Discs Large Homolog 1 (DLG1). *J. Biol. Chem.* 286, 43559-43568. 10.1074/jbc.M111.294603 22027822 PMC3234831

[JCS259984C56] O'Shaughnessy, E. M., Duffy, W., Garcia-Vega, L., Hussey, K., Burden, A. D., Zamiri, M. and Martin, P. E. (2021). Dysregulation of connexin expression plays a pivotal role in psoriasis. *Int. J. Mol. Sci.* 22, 6060. 10.3390/ijms22116060 34199748 PMC8200029

[JCS259984C57] Palatinus, J. A., Rhett, J. M. and Gourdie, R. G. (2012). The connexin43 carboxyl terminus and cardiac gap junction organization. *Biochim. Biophys. Acta Biomembr.* 1818, 1831-1843. 10.1016/j.bbamem.2011.08.006 PMC3244566 21856279

[JCS259984C58] Reuver, S. M. and Garner, C. C. (1998). E-cadherin mediated cell adhesion recruits SAP97 into the cortical cytoskeleton. *J. Cell Sci.* 111, 1071-1080. 10.1242/jcs.111.8.1071 9512503

[JCS259984C59] Rhett, J. M., Jourdan, J. and Gourdie, R. G. (2010). Connexin 43 connexon to gap junction transition is regulated by zonula occludens-1. *Mol. Biol. Cell* 22, 1516-1528. 10.1091/mbc.e10-06-0548 PMC3084674 21411628

[JCS259984C60] Rivera, C., Yamben, I. F., Shatadal, S., Waldof, M., Robinson, M. L. and Griep, A. E. (2009). Cell-autonomous requirements for Dlg-1 for lens epithelial cell structure and fiber cell morphogenesis. *Dev. Dyn.* 238, 2292-2308. 10.1002/dvdy.22036 19623611 PMC3016059

[JCS259984C61] Saidi Brikci-Nigassa, A., Clement, M.-J., Ha-Duong, T., Adjadj, E., Ziani, L., Pastre, D., Curmi, P. A. and Savarin, P. (2012). Phosphorylation controls the interaction of the connexin43 C-terminal domain with tubulin and microtubules. *Biochemistry* 51, 4331-4342. 10.1021/bi201806j 22558917

[JCS259984C62] Salat-Canela, C., Sesé, M., Peula, C., Ramón y Cajal, S. and Aasen, T. (2014). Internal translation of the connexin 43 transcript. *Cell Commun. Signal.* 12, 31. 10.1186/1478-811X-12-31 24884945 PMC4108066

[JCS259984C63] Saraceno, C., Marcello, E., Di Marino, D., Borroni, B., Claeysen, S., Perroy, J., Padovani, A., Tramontano, A., Gardoni, F. and Di Luca, M. (2014). SAP97-mediated ADAM10 trafficking from Golgi outposts depends on PKC phosphorylation. *Cell Death Dis.* 5, e1547. 10.1038/cddis.2014.492 25429624 PMC4260750

[JCS259984C64] Shaw, R. M., Fay, A. J., Puthenveedu, M. A., Von Zastrow, M., Jan, Y.-N. and Jan, L. Y. (2007). Microtubule plus-end-tracking proteins target gap junctions directly from the cell interior to adherens junctions. *Cell* 128, 547-560. 10.1016/j.cell.2006.12.037 17289573 PMC1955433

[JCS259984C65] Singh, D. and Lampe, P. D. (2003). Identification of connexin-43 interacting proteins. *Cell Commun. Adhes.* 10, 215-220. 10.1080/cac.10.4-6.215.220 14681019

[JCS259984C66] Smyth, J. W., Vogan, J. M., Buch, P. J., Zhang, S.-S., Fong, T. S., Hong, T.-T. and Shaw, R. M. (2012). Actin cytoskeleton rest stops regulate anterograde traffic of connexin 43 vesicles to the plasma membrane. *Circ. Res.* 110, 978-989. 10.1161/CIRCRESAHA.111.257964 22328533 PMC3621031

[JCS259984C67] Solan, J. L. and Lampe, P. D. (2014). Specific Cx43 phosphorylation events regulate gap junction turnover in vivo. *FEBS Lett.* 588, 1423-1429. 10.1016/j.febslet.2014.01.049 24508467 PMC3989505

[JCS259984C68] Sorgen, P., Duffy, H. S., Sahoo, P., Coombs, W., Delmar, M. and Spray, D. C. (2004). Structural changes in the carboxyl terminus of the gap junction protein connexin43 indicates signaling between binding domains for c-Src and zonula occludens-1. *J. Biol. Chem.* 279, 54695-54701. 10.1074/jbc.M409552200 15492000

[JCS259984C69] Stauch, K., Kieken, F. and Sorgen, P. (2012). Characterization of the structure and intermolecular interactions between the connexin 32 carboxyl-terminal domain and the protein partners synapse-associated protein 97 and calmodulin. *J. Biol. Chem.* 287, 27771-27788. 10.1074/jbc.M112.382572 22718765 PMC3431650

[JCS259984C70] Stephens, R., Lim, K., Portela, M., Kvansakul, M., Humbert, P. O. and Richardson, H. E. (2018). The scribble cell polarity module in the regulation of cell signaling in tissue development and tumorigenesis. *J. Mol. Biol.* 430, 3585-3612. 10.1016/j.jmb.2018.01.011 29409995

[JCS259984C71] Su, V. and Lau, A. F. (2014). Connexins: Mechanisms regulating protein levels and intercellular communication. *FEBS Lett.* 588, 1212-1220. 10.1016/j.febslet.2014.01.013 24457202 PMC3989427

[JCS259984C72] Subbaiah, V. K., Kranjec, C., Thomas, M. and Banks, L. (2011). PDZ domains: the building blocks regulating tumorigenesis. *Biochem. J.* 439, 195-205. 10.1042/BJ20110903 21954943

[JCS259984C73] Sun, P., Dong, L., Macdonald, A. I., Akbari, S., Edward, M., Hodgins, M. B., Johnstone, S. R. and Graham, S. V. (2015). HPV16 E6 controls the gap junction protein Cx43 in cervical tumour cells. *Viruses* 7, 5243-5256. 10.3390/v7102871 26445057 PMC4632379

[JCS259984C74] Theiss, C. and Meller, K. (2002). Aluminum impairs gap junctional intercellular communication between astroglial cells in vitro. *Cell Tissue Res.* 310, 143-154. 10.1007/s00441-002-0639-3 12397369

[JCS259984C75] Thomas, T., Jordan, K. and Laird, D. W. (2001). Role of cytoskeletal elements in the recruitment of Cx43-GFP and Cx26-YFP into gap junctions. *Cell Commun. Adhes.* 8, 231-236. 10.3109/15419060109080729 12064594

[JCS259984C76] Thomas, M., Dasgupta, J., Zhang, Y., Chen, X. and Banks, L. (2008). Analysis of specificity determinants in the interactions of different HPV E6 poteins with their PDZ domain-containing substrates. *Virology* 376, 371-378. 10.1016/j.virol.2008.03.021 18452965

[JCS259984C77] Totland, M. Z., Rasmussen, N. L., Knudsen, L. M. and Leithe, E. (2020). Regulation of gap junction intercellular communication by connexin ubiquitination: physiological and pathophysiological implications. *Cell. Mol. Life Sci.* 77, 573-591. 10.1007/s00018-019-03285-0 31501970 PMC7040059

[JCS259984C78] Toyofuku, T., Yabuki, M., Otsu, K., Kuzuya, T., Hori, M. and Tada, M. H. (1998). Direct association of the gap junction protein connexin-43 with ZO-1 in cardiac myocytes. *J. Biol. Chem* 273, 12725-12731. 10.1074/jbc.273.21.12725 9582296

[JCS259984C79] Underhill, S. M., Wheeler, D. S. and Amara, S. G. (2015). Differential regulation of two isoforms of the glial glutamate transporter EAAT2 by DLG1 and CaMKII. *J. Neurosci.* 35, 5260-5270. 10.1523/JNEUROSCI.4365-14.2015 25834051 PMC4380999

[JCS259984C80] Valdebenito, S., Barreto, A. and Eugenin, E. A. (2018). The role of connexin and pannexin containing channels in the innate and acquired immune response. *Biochim. Biophys. Acta Biomembr.* 1860, 154-165. 10.1016/j.bbamem.2017.05.015 28559189 PMC6065251

[JCS259984C81] Won, S., Levy, J. M., Nicoll, R. A. and Roche, K. W. (2017). MAGUKs: multifaceted synaptic organizers. *Curr. Opin. Neurobiol.* 43, 94-101. 10.1016/j.conb.2017.01.006 28236779 PMC5447471

[JCS259984C82] Woods, D. F., Hough, C., Peel, D., Callaini, G. and Bryant, P. J. (1996). Dlg protein is required for junction structure, cell polarity, and proliferation control in Drosophila epithelia. *J. Cell Biol.* 134, 1469-1482. 10.1083/jcb.134.6.1469 8830775 PMC2120992

[JCS259984C83] Wu, H., Reuver, S. M., Kuhlendahl, S., Chung, W. J. and Garner, C. C. (1998). Subcellular targeting and cytoskeletal attachment of SAP97 to the epithelial lateral membrane. *J. Cell. Sci.* 111, 2365-2376. 10.1242/jcs.111.16.2365 9683631

[JCS259984C84] Ye, F., Zeng, M. and Zhang, M. (2018). Mechanisms of MAGUK-mediated cellular junctional complex organization. *Curr. Opin. Struct. Biol.* 48, 6-15. 10.1016/j.sbi.2017.08.006 28917202

[JCS259984C85] Zeitz, M. J., Calhoun, P. J., James, C. C., Taetzsch, T., George, K. K., Robel, S., Valdez, G. and Smyth, J. W. (2019). Dynamic UTR usage regulates alternative translation to modulate gap junction formation during stress and aging. *Cell Rep.* 27, 2737-2747.e2735. 10.1016/j.celrep.2019.04.114 31141695 PMC6857847

[JCS259984C86] Zheng, L., Li, H., Cannon, A., Trease, A. J., Spagnol, G., Zheng, H., Radio, S., Patel, K., Batra, S. and Sorgen, P. L. (2019). Phosphorylation of Cx43 residue Y313 by Src contributes to blocking the interaction with Drebrin and disassembling gap junctions. *J. Mol. Cell. Cardiol.* 126, 36-49. 10.1016/j.yjmcc.2018.11.008 30448479 PMC8961861

