# Supplementary material for: The human discs large protein 1 interacts with and maintains connexin 43 at the plasma membrane in keratinocytes
Source: J Cell Sci. 2023 Jun 8;136(11):jcs259984. doi: 10.1242/jcs.259984 (PMC10309592; doi:10.1242/jcs.259984)
Supplement: Supplementary information [file joces-136-259984-s1.pdf]

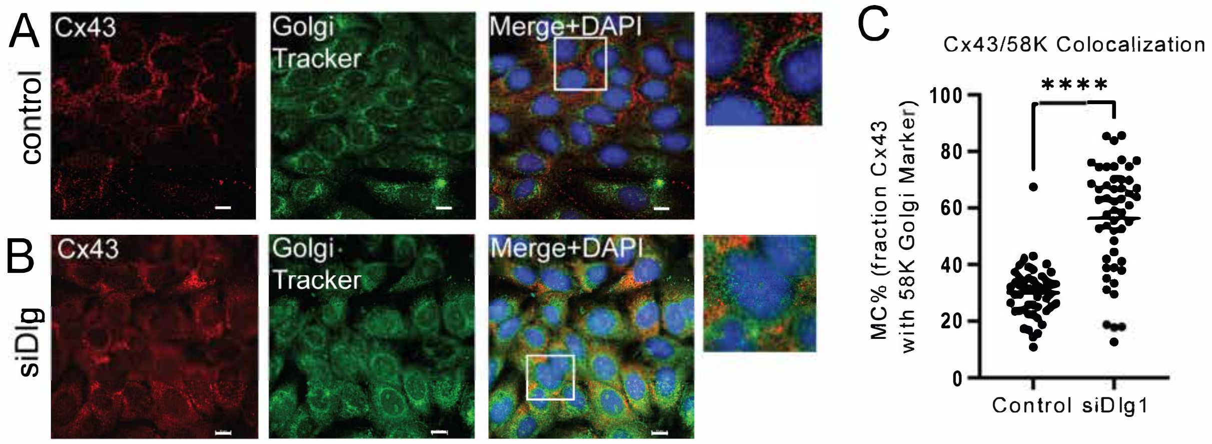

Fig. S1. Cx43 colocalisation with Golgi Tracker. Cx43 (red) antibody and Golgi Tracker staining (green) of HaCaT cells treated with A. control siRNA or B. with siRNA against Dlg1 (siDlg). Merge +DAPI, merged images of red and green channels plus DAPI staining in blue to visualise nuclei. Enlarged sections of each merged image {white boxed areas) are shown on the right-hand side. Bar = 10 μm. C. Manders' co-efficients (MC) measurement of co-occurrence of Cx43 and Golgi staining. 50 individual cells were analysed for each treatment group. ****$p<0.0001$.

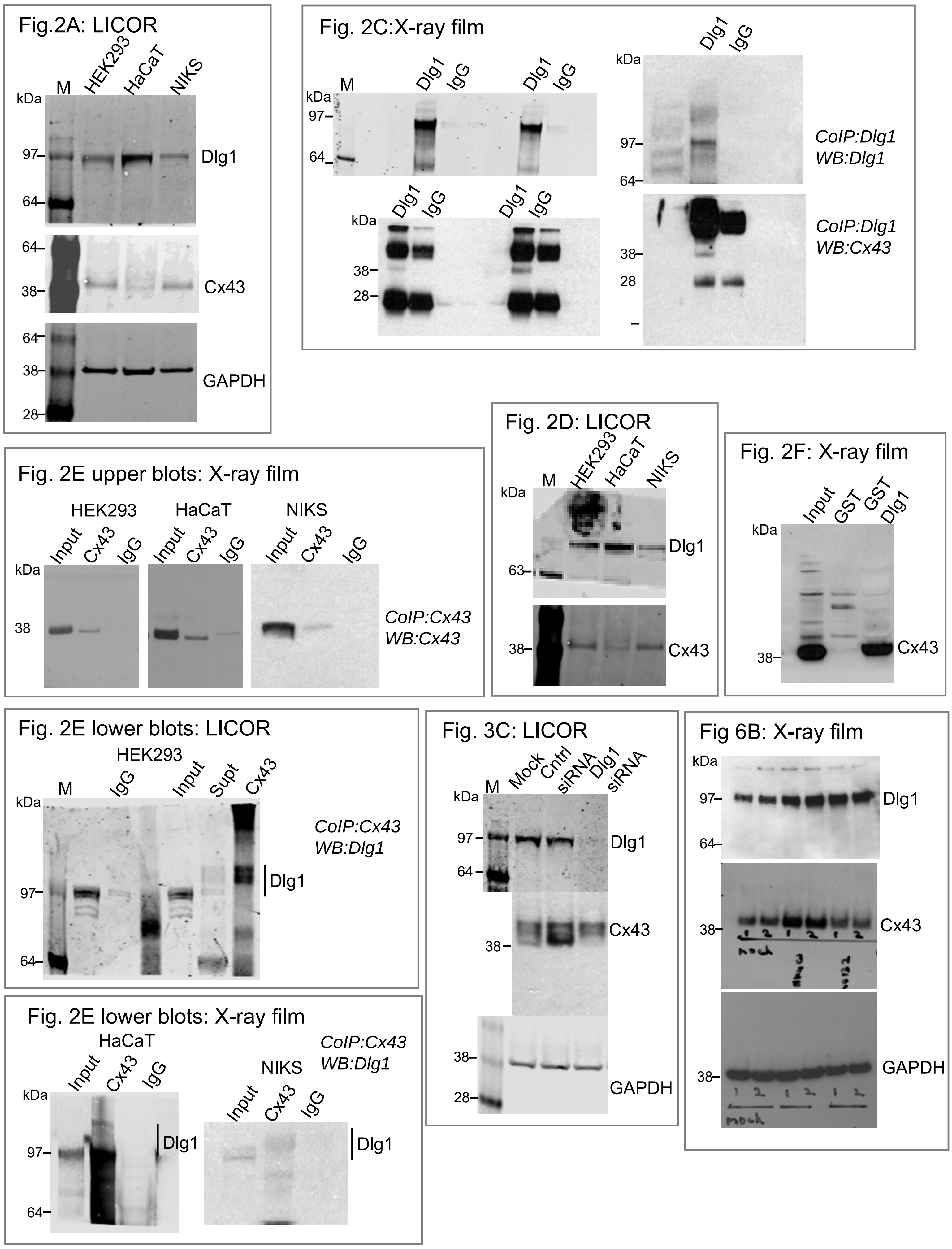

Fig. S2. Western blot transparency. Larger portions of the images of all western blots shown in the figures together with statements of how the images were produced.
