# Supplementary material for: The human discs large protein 1 interacts with and maintains connexin 43 at the plasma membrane in keratinocytes
Source: J Cell Sci. 2023 Jun 8;136(11):jcs259984. doi: 10.1242/jcs.259984 (PMC10309592; doi:10.1242/jcs.259984)
Supplement: Supplementary file 1 [file joces-136-259984_review_history.pdf]

The human discs large protein interacts with and maintains connexin 43 at the plasma membrane in keratinocytes

Harry Scott, Li Dong, Andrew Stevenson, Alasdair I. MacDonald, Sharmila Srinivasan, Paola Massimi, Lawrence Banks, Patricia E. Martin, Scott R. Johnstone and Sheila V. Graham

DOI: 10.1242/jcs.259984

Editor: Kathleen Green

Review timeline

Original submission:	8 March 2022
Editorial decision:	9 May 2022
First revision received:	26 November 2022
Editorial decision:	19 December 2022
Second revision received:	1 February 2023
Editorial decision:	2 March 2023
Third revision received:	20 April 2023
Accepted:	21 April 2023

Original submission

First decision letter

MS ID#: JOCES/2022/259984

MS TITLE: The human Discs large protein (Dlg1) controls Connexin 43 (Cx43) trafficking to the plasma membrane and gap junctional communication in keratinocytes.

AUTHORS: Li Dong, Harry Scott, Sharmila Srinivasan, Andrew Stevenson, Alasdair I MacDonald, Paola Massimi, Lawrence Banks, Patricia E Martin, Scott R Johnstone, and Sheila V Graham
ARTICLE TYPE: Research Article

We have now reached a decision on the above manuscript.

To see the reviewers' reports and a copy of this decision letter, please go to: https://submit-jcs.biologists.org and click on the 'Manuscripts with Decisions' queue in the Author Area. (Corresponding author only has access to reviews.)

As you will see, the reviewers raise a number of substantial criticisms that prevent me from accepting the paper at this stage. They suggest, however, that a revised version might prove acceptable, if you can address their concerns. If you think that you can deal satisfactorily with the criticisms on revision, I would be pleased to see a revised manuscript. We would then return it to the reviewers.

Please ensure that you clearly highlight all changes made in the revised manuscript. Please avoid using 'Tracked changes' in Word files as these are lost in PDF conversion.

I should be grateful if you would also provide a point-by-point response detailing how you have dealt with the points raised by the reviewers in the 'Response to Reviewers' box. Please attend to all of the reviewers' comments. If you do not agree with any of their criticisms or suggestions please explain clearly why this is so.

© 2023. Published by The Company of Biologists under the terms of the Creative Commons Attribution License (https://creativecommons.org/licenses/by/4.0/).

Reviewer 1

Advance summary and potential significance to field

This manuscript by Dong and colleagues investigates the functional role of the previously reported interaction between the scaffolding protein, human discs large protein (Dlg1) and Cx43 in previously uninvestigated keratinocytes. The authors provide evidence that this protein-protein interaction occurs in keratinocytes and epithelial tissue. They use Dlg1 knockdown strategies to show that this does not alter the transcript levels of Cx43 but leads to a reduction in Cx43, an increase in Cx43 levels in the Golgi apparatus, and an overall reduction in gap junction intercellular communication as revealed by dye transfer. The authors provide data supporting a mechanism where Dlg1 facilitates Cx43 trafficking and assembly into gap junctions in keratinocytes.

This study builds on the previously reported direct interaction of Dlg1 and Cx43 in NRK cells and HPV-infected cervical tumor cells. While the interaction of Dlg1 and Cx43 has been documented best in E6 oncoprotein expressing cells, the current study establishes that E6 is not necessary for this interaction to occur in keratinocytes. Importantly, the authors examine the cellular consequences of this interaction in regulating Cx43 trafficking and assembly into functional gap junction.

Comments for the author

While the premise of the study is interesting, the image quality is great and there is potential for a strong contribution here, there are significant issues that require attention in order to strengthen and clarify the overarching story.

1) The authors provide evidence that Dlg1 and Cx43 interact in native epithelial tissue base on the proximity ligation assay data presented in Figure 1. However, given the limitations of the experiments as described, the data is restricted by only providing evidence that Dlg1 and Cx43 are close to each other (40nm). At the molecular level, 40 nm is the equivalent of the thickness of 4 lipid bilayers, so this does not solidify a protein-protein interaction but rather supports the premise. I suspect that Dlg1 and Cx43 do indeed interact in epithelial tissue and keratinocytes, but this experiment alone does not prove this point and caution needs to be used when describing this experiment. Also, the PLA is noted at 40 um in the figure legend which I assume is a mistake. 2ndry is not defined in the figure legend? Can better labeling controls be used as simply leaving out the primary antibodies is not a strong control?

2) The colocalization of Dlg1 and Cx43 in the three cell types shown in figure 2 is convincing. However, the data in Figure 2F is incomplete and needs to be presented better as it appears as if a band is being cropped in half (asterisks). This is defined as a nonspecific band due to antibody cross reacting with another protein. Given that many excellent antibodies to Cx43 exist that have no significant cross reactivity, can this be improved? Importantly the authors are immunoprecipitating with anti-Dlg1, but they do not show the back blot where they immunolabel for Dlg1 in addition to Cx43. Conversely, in panel G, they IP for Cx43 but do not show the back blot for both Cx43 and Dlg1. Why is only HaCaT shown in panel G and also not HEK 293 and NIKS cells as it appears the antibodies work on all three cell types?

3) In figure 3, the authors should include the data from Figure 6 A-C to show the quantification of the Dlg knockdown and the quantified effect on Cx43. Alternatively, this figure could be moved ahead of Figure 3. In Figure 3, gels need to include molecular weight standards. The staining profile of Cx43 following Dlg1 knockdown is very vesicular looking near the nucleus and not the traditional lamella pattern of Cx43 typically seen when Cx43 is localized to the Golgi apparatus. The Golgi staining dye is not particularly convincing with lots of background. The authors should confirm these findings by double labeling for a resident protein of the Golgi apparatus and also consider that the vesicle-like Cx43-positive structures are sites of lysosomes or late endosomes which tend to accumulate near the Golgi apparatus.

4) The authors should refer to the data in Figure 4 as a knockdown of Dlg1 and not the absence of Dlg1 as stated.

© 2023. Published by The Company of Biologists under the terms of the Creative Commons Attribution License (https://creativecommons.org/licenses/by/4.0/).

5) It is not clear to this reviewer how knockdown of Dlg1 increased Cx43 degradation from the data present. In mock treated cells compared to siDlg knockdown cells the levels of Cx43 are reduced by about 30-40% (Figure 5) but there is no quantification to know if this is significant. When lysosomes are inhibited, all cells show a substantial increase in total Cx43 regardless of whether Dlg1 was reduced by siRNA transfection (panels D and E). While I understand that the fold increase in Cx43 is higher in lysosome inhibited cells where Dlg1 is reduced but the starting levels of Cx43 are also different across these experiments so can one directly compare the fold changes of 2.8 to 1.7 as stated in the results? In the event there is a difference in Cx43 degradation in Dlg1 reduced cells, can the authors elaborate on the mechanism at play? Does Cx43 reach the cell surface and immediately get targeted to the lysosomes? Does trafficking deficient Cx43 get delivered directly to lysosomes? How would Dlg1 selectively participate in these pathways?

6) The functional data in figure 6 is convincing but needs to be quantified. Magnification bars are missing. The position of the region of interest should be maintained in each field of view within an experimental set.

7) The authors need to provide a model of how Dlg1 acts to both cause a reservoir of Cx43 in the Golgi and also regulate its lysosomal degradation.

8) There are several typos, spelling and editorial mistakes that need to be corrected.

Overall, this is a potentially interesting story that needs to be enhanced and clarified.

Reviewer 2

Advance summary and potential significance to field

In this manuscript, Dong et al. provide evidence that the MAGUK family scaffolding protein hDlg1 aids Cx43 delivery to the plasma membrane (PM) in keratinocytes. While these findings are new and interesting, many open questions remain unanswered, and the manuscript would greatly benefit from a number of additions:

Comments for the author

In this manuscript, Dong et al. provide evidence that the MAGUK family scaffolding protein hDlg1 aids Cx43 delivery to the plasma membrane (PM) in keratinocytes. While these findings are new and interesting, many open questions remain unanswered, and the manuscript would greatly benefit from a number of additions:
- Is the Cx43/Dlg1 interaction keratinocyte specific, and if yes, why may Cx43 require Dlg1 in keratinocytes but not in other cell types for efficient PM delivery? This should be discussed.
- Dlg1 has been found previously to interact with another connexin, Cx32 (Duffy et al.). These findings should be mentioned and related to the Cx43/Dlg1 interaction.
- Do the authors think Cx43 interacts with its C-terminal amino acid residues with the PDZ domain of Dlg1 similar to ZO-1? If yes, how is Dlg1/ZO-1 binding competition regulated? Some discussion would be appropriate.
- PM Cx43 levels in wt and Dlg1 KD cells need to be quantified. How strongly is Cx43 delivery to the PM reduced when Dgl1 is knocked down? From the images, the % reduction in PM Cx43 in the KD cells is not clear.
- Figures 1 and 3 would greatly benefit from quantifications. Also, in Figure 1, showing single channel images in addition to the merged images would allow a better visualization of Cx43 and Dlg1 in the tissue cells.
- The Dlg1 and especially the Cx43 signal in Figures 2 and 3 is hard to see. Presenting single channel images (green, red) in b/w may improve image quality.
- Dlg1 KD: Figures 3C and 6A are redundant. 6B, C would fit much better to 3C. Also, why was the Dlg1 KD image in Figure 2B enhanced? It makes it hard to appreciate the 84% efficiency of the knockdown.
- Dye transfer assays (Figure 6D) should be quantified. Also, why is there no dye transfer at all if some GJs remain in the PM in the Dlg1 KD as evident from Figure 3B-G?

© 2023. Published by The Company of Biologists under the terms of the Creative Commons Attribution License (https://creativecommons.org/licenses/by/4.0/).

Additional minor points:
- There is no Figure 4C.
- Results header: Dlg1 controls Cx43 membrane location(in) normal keratinocytes
- Results header: Cx43 membrane localisation and gap junctional communication (is) regulated by Dlg1
- Discussion, para starting with Zonula Occludens-1: spelling of data in line 3
- P. 18, l. 5: loss of intracellular cellular communication
- Figures 5A and 6C are redundant.
- si and sh and Dlg and Dlg1 are used inconsistently in Figures 5 and 6.
- Adding line numbers would help in the review process.

Reviewer 3

Advance summary and potential significance to field

The manuscript entitled “The human Disc large protein (DIg1) controls Connexin43 trafficking to the plasma membrane and gap junctional communication in keratinocytes” demonstrates that the loss of Dig1 in keratinocytes reduces Cx43 at the plasma membrane accompanied by a reduction in cell-to-cell communication. The authors demonstrate this fact by using a human keratinocytes cell line and a spontaneously immortalized human foreskin fibroblast.

Comments for the author

The manuscript is very well written, and it is easy to follow, however there are several points that should be addressed by the authors:

Introduction:
- The authors should avoid abbreviations if the full name is not described at least once in the text. Please correct accordingly throughout the introduction.

Methods:
- A paragraph should be included to explain the statistical analysis used in the manuscript.

Results and figure legends:
- High magnification images from figures 1, 2 and 3 should be changed, the authors should show a 100X magnification image from a confocal microscope.
- Figure 1C. When the authors show the results from the PLA, it seems that Cx43 and Dig1 are localized at the nuclei, however images from Figure 1B do not show the same localization. Can the authors explain that?
- Western-blot from Figure 2D demonstrates that the higher Cx43 levels are present in HEK293 and NIKS, however these data do not correlate with the IF images showed in Figure 2A. Please explain. Also, why the authors decided to work with HaCaT cells if they have the lower Cx43 levels according to the western-blot in Figure 2D?
- Statistics should be added to Figure 2E.
- What happen with Cx43 in Dig1-IP experiments in Figure 2G?
- Molecular weight should be added to western-blot n Figure 3C.
- Figure 4 should be revised by the authors. Figure legend does nor correlate with the data presented.
- HaCaT cells treated with NH_4Cl, increase Dig1 and Cx43 protein levels after 4 and 8 hours incubation (Fig 5C). These data are not shown in Mock and siRNa control in Figure 5C. Can the authors explain this fact?
- Number of experiments and number of replicates/experiments should be added in each figure legend.

© 2023. Published by The Company of Biologists under the terms of the Creative Commons Attribution License (https://creativecommons.org/licenses/by/4.0/).

First revision

Author response to reviewers' comments

We would like to thank the reviewers for their extremely helpful comments that have allowed us to thoroughly revise our paper. We are very pleased that the reviewers believe our work is new and interesting. We apologise for the length of time taken to revise our manuscript. This was due to a three month wait for permission to harvest fresh cutaneous skin and a serious personal problem in the life of the senior author. We believe this new submission is much improved and we hope it provides clarification of the story. We have revised all but one figure (Figure 4, now Figure 5) and included Figure 8 presenting a summary of our data and a model for the Cx43 life cycle upon depletion of Dlg 1. All the revision experiments were carried out by author Scott because author Dong had left the lab. Scott's contribution to the paper is now far greater than that of Dong therefore we have now listed Scott as first author. We detail below a point- by-point response to comments.

Reviewer 1 Comments for the Author...

1) The authors provide evidence that Dlg 1 and Cx43 interact in native epithelial tissue base on the proximity ligation assay data presented in Figure 1. However, given the limitations of the experiments as described, the data is restricted by only providing evidence that Dlg 1 and Cx43 are close to each other (40nm). At the molecular level, 40 nm is the equivalent of the thickness of 4 lipid bilayers, so this does not solidify a protein-protein interaction but rather supports the premise. I suspect that Dlg 1 and Cx43 do indeed interact in epithelial tissue and keratinocytes, but this experiment alone does not prove this point and caution needs to be used when describing this experiment. Also, the PLA is noted at 40 um in the figure legend which I assume is a mistake. 2ndry is not defined in the figure legend? Can better labeling controls be used as simply leaving out the primary antibodies is not a strong control?

We agree that PLA does not prove a direct interaction between proteins. Our previous studies (MacDonald et al, 2012: doi: 10.1042/BJ20111144) showed a direct interaction in vitro between FLAG-tagged Cx43 and GST-tagged Dlg1. Those data together with co- immunoprecipitation, GST pull down experiments and colocalization in confocal immunofluorescence microscopy experiments in this manuscript and in our previous work are strongly suggestive of a protein-protein interaction. We have replaced the previous PLA data in archival formalin fixed paraffin embedded tissues with new confocal immunofluorescence microscopy experiments in fresh human cutaneous skin. We show colocalization of Cx43 in the epithelial and dermal layers and in adipose cells in the same tissues.

2) The colocalization of Dlg 1 and Cx43 in the three cell types shown in figure 2 is convincing. However, the data in Figure 2F is incomplete and needs to be presented better as it appears as if a band is being cropped in half (asterisks). This is defined as a nonspecific band due to antibody cross reacting with another protein. Given that many excellent antibodies to Cx43 exist that have no significant cross reactivity, can this be improved?

We have replaced the cropped images with full images in Figure 2C. There is only one antibody against Dlg1 (H60) that can be used in coimmunoprecipitation, and unfortunately it is no longer in production. This antibody brings down a large amount of immunoglobulin which reacts with the Cx43 antibody on western blots. Previously we were able to use the CleanBlot system (Thermo Scientific) to get rid of the antibody bands (and to prove they were antibody bands: see MacDonald et al: doi: 10.1042/BJ20111144 Figure 5A and supplementary Figure S1). Unfortunately, we were unable to take this approach again to repeat the Co-IPs because the CleanBlot system requires film development using an X-ray processor, which is no longer available to us, and LI-COR imaging is not compatible with CleanBlot. This means that the CoIP:Dlg1, WB:Cx43 full images in Figure 2C now show the antibody bands (labelled ab). This also precluded inclusion of the input bands on the same blot since the input bands were not sufficiently visible on the co-immunoprecipitation blots due to the density of the antibody

© 2023. Published by The Company of Biologists under the terms of the Creative Commons Attribution License (https://creativecommons.org/licenses/by/4.0/).

bands precluding longer exposure of the blots.

Importantly the authors are immunoprecipitating with anti-Dlgl, but they do not show the back blot where they immunolabel for Dlg 1 in addition to Cx43. Conversely, in panel G, they IP for Cx43 but do not show the back blot for both Cx43 and Dlg 1. Why is only HaCaT shown in panel G and also not HEK 293 and NIKS cells as it appears the antibodies work on all three cell types?

We have now also shown in Figure 2C the CoIP:Cx43, WB:Dlg1 experiment for all three cell lines. Back blots are included for each set of experiments. We apologise for missing these out in the first submission. We now show a GST pull down for HaCaT cells but not for other cells. This is because all the subsequent experiments in the manuscript use HaCaT cells and we have previously shown GST pull downs in other epithelial cells (MacDonald et al: doi: 10.1042/BJ20111144 Figure 5).

3) In figure 3, the authors should include the data from Figure 6 A-C to show the quantification of the Dlg knockdown and the quantified effect on Cx43. Alternatively, this figure could be moved ahead of Figure 3. In Figure 3, gels need to include molecular weight standards.

We agree with the reviewer and have reorganization the figures. Figure 3 now shows graphs of Dlg1 and Cx43 levels in control and Dlg1 siRNA-treated cells. The gel images have been minimized to sit in the graphs and size markers are now included.

The staining profile of Cx43 following Dlg 1 knockdown is very vesicular looking near the nucleus and not the traditional lamella pattern of Cx43 typically seen when Cx43 is localized to the Golgi apparatus. The Golgi staining dye is not particularly convincing with lots of background. The authors should confirm these findings by double labeling for a resident protein of the Golgi apparatus and also consider that the vesicle-like Cx43- positive structures are sites of lysosomes or late endosomes which tend to accumulate near the Golgi apparatus.

We have repeated the Golgi experiment using 58K as a resident Golgi protein and included new images in Figure 4. A very similar percentage colocalization of Cx43 with 58K was found compared to the previous data using Golgi Tracker (now supplementary Figure 1). New quantification of Cx43 location in the lysosomes showed only a small amount of Cx43 present there but upon Dlgl depletion there was a 50% increase in colocalization of Cx43 with LAMP2 (Figure 6E). We have included a statement about Golgi-adjacent lysosomes in the Discussion on line 285.

3) The authors should refer to the data in Figure 4 as a knockdown of Dlgl and not the absence of Dlg 1 as stated.

We apologise for this error and have corrected it.

4) It is not clear to this reviewer how knockdown of Dlg 1 increased Cx43 degradation from the data present. In mock treated cells compared to siDlg knockdown cells the levels of Cx43 are reduced by about 30-40% (Figure 5) but there is no quantification to know if this is significant.

We have now added in the quantification, and it is shown in Figure 6C.

When lysosomes are inhibited, all cells show a substantial increase in total Cx43 regardless of whether Dlg 1 was reduced by siRNA transfection (panels D and E). While I understand that the fold increase in Cx43 is higher in lysosome inhibited cells where Dlg 1 is reduced but the starting levels of Cx43 are also different across these experiments so can one directly compare the fold changes of 2.8 to 1.7 as stated in the results?

We agree with the reviewer and have removed the statement about comparisons and have added in to Figure 6 (formerly Figure 5) an experiment to quantify Cx43 colocalisation with lysosomal

© 2023. Published by The Company of Biologists under the terms of the Creative Commons Attribution License (https://creativecommons.org/licenses/by/4.0/).

marker LAMP2 by Manders' colocalisation coefficient.

In the event there is a difference in Cx43 degradation in Dlg 1 reduced cells, can the authors elaborate on the mechanism at play? Does Cx43 reach the cell surface and immediately get targeted to the lysosomes? Does trafficking deficient Cx43 get delivered directly to lysosomes? How would Dlg 1 selectively participate in these pathways?

We have quantified the relative increase of Cx43 in the lysosomes upon Dlg1 depletion and find a 50% increase. However, at steady state very little Cx43 appears to be located in the lysosomes overall. This would argue that if trafficking-deficient Cx43 is recycled to the lysosomes from the ER/Golgi then degradation is very speedy. However, there are alternative explanations. We have explored these in the Discussion but feel that it would take a significant length of time to gather experimental evidence to support or refute the various alternatives and this is outside the scope of the current manuscript.

6) The functional data in figure 6 is convincing but needs to be quantified. Magnification bars are missing. The position of the region of interest should be maintained in each field of view within an experimental set.

We decided that the dye transfer experiment in Figure 6 was suboptimal for quantification, so we have carried out a new parachute assay and quantified loss of gap junctional communication upon Dlg1 depletion and included new controls (Figure 7 formerly Figure 6).

7) The authors need to provide a model of how Dlg 1 acts to both cause are servoir of Cx43 in the Golgi and also regulate its lysosomal degradation.

We have written a new Discussion section (starting at line 269) and included a model of our data (Figure 8) to try to address this comment.

8) There are several typos, spelling and editorial mistakes that need to be corrected.

We apologise for these mistakes. Hopefully we have corrected these now.

Reviewer 2 Comments for the Author...

In this manuscript, Dong et al. provide evidence that the MAGUK family scaffolding protein hDlg1 aids Cx43 delivery to the plasma membrane (PM) in keratinocytes. While these findings are new and interesting, many open questions remain unanswered, and the manuscript would greatly benefit from a number of additions:

- Is the Cx43/Dlg1 interaction keratinocyte specific, and if yes, why may Cx43 require Dlg 1 in keratinocytes but not in other cell types for efficient PM delivery? This should be discussed.

No, we don't believe that the interaction is specific to keratinocytes. Now we have shown interaction in vivo in dermal cells and in adipocytes (Figure 1).

- Dlg 1 has been found previously to interact with another connexin, Cx32 (Duffy et al.). These findings should be mentioned and related to the Cx43/Dlg1 interaction.

We apologise for missing out this important information. In fact, we have used Cx32 as a control for our interaction previously (see MacDonald et al: doi: 10.1042/BJ20111144). We have now referenced Duffy et al 2007 and Stauch et al, 2012 and included two new sections discussing Cx32 starting on lines 94 and 315.

- Do the authors think Cx43 interacts with its C-terminal amino acid residues with the PDZ domain of Dlg 1 similar to ZO-1? If yes, how is Dlg1/ZO-1 binding competition regulated? Some discussion would be appropriate.

© 2023. Published by The Company of Biologists under the terms of the Creative Commons Attribution License (https://creativecommons.org/licenses/by/4.0/).

We have already published that the interaction of Cx43 with Dlg1 is different to that with ZO-1 (see MacDonald et al: doi: 10.1042/BJ20111144). The last five amino acids of Cx43 are not required to bind Dlg1 and Cx43 does not bind the central PDZ domain of Dlg1 in comparison to ZO-1, which does. We believe that the Cx43/Dlg1 interaction is more like the Cx32 /Dlg interaction and are currently carrying out experiments to elucidate this.

- PM Cx43 levels in wt and Dlg 1 KD cells need to be quantified. How strongly is Cx43 delivery to the PM reduced when Dgl1 is knocked down? From the images, the % reduction in PM Cx43 in the KD cells is not clear.

We agree with the reviewer and have now quantified the reduction in plasma membrane Cx43 upon Dlg1 knock down. The new graph is shown in Figure 4C.

- Figures 1 and 3 would greatly benefit from quantifications. Also, in Figure 1, showing single channel images in addition to the merged images would allow a better visualization of Cx43 and Dlg 1 in the tissue cells.

We have created a new Figure 1. For the in vivo tissue we now use freshly collected cutaneous skin and show single channels as well as merged images. We have quantified the reduction in plasma membrane Cx43 upon Dlg1 knock down. The new graph is shown in Figure 4C. We previously included Manders' colocalization co-efficient quantifications of Cx43 colocalisation with ER and Golgi markers. Now we also include quantification of Cx43 and Dlg levels upon control and Dlg1 siRNA knock down.

- The Dlg 1 and especially the Cx43 signal in Figures 2 and 3 is hard to see. Presenting single channel images (green, red) in b/w may improve image quality.

Unfortunately, switching to black and white images did not significantly improve the image brightness. Since the one reviewer did not comment negatively on the images and the other reviewer praised the images, we prefer to leave them as is.

- Dlg 1 KD: Figures 3C and 6A are redundant. 6B, C would fit much better to 3C. Also, why was the Dlg 1 KD image in Figure 2B enhanced? It makes it hard to appreciate the 84% efficiency of the knockdown.

We have taken the reviewer's helpful suggestion and changed the figures accordingly.
The reason for enhancement of Dlg1 was that we felt it was important to show that residual Dlg1 remained at the plasma membrane.

- - Dye transfer assays (Figure 6D) should be quantified. Also, why is there no dye transfer at all if some GJs remain in the PM in the Dlg 1 KD as evident from Figure 3B-G?

We would like to thank the reviewer for this point. On reflection we felt that this dye transfer experiment was suboptimal. We have now carried out a parachute assay and quantified the data (Figure 7).

Additional minor points:
- There is no Figure 4C.
- Results header: Dlg 1 controls Cx43 membrane location (in) normal keratinocytes
- Results header: Cx43 membrane localisation and gap junctional communication (is) regulated by Dlg 1
- Discussion, para starting with Zonula Occludens-1: spelling of data in line 3
- P. 18, l. 5: loss of intracellular cellular communication
- Figures 5A and 6C are redundant.
- si and sh and Dlg and Dlg 1 are used inconsistently in Figures 5 and 6.
- Adding line numbers would help in the review process.

We would like to thank the reviewer for pointing out these errors, which we have now corrected.

© 2023. Published by The Company of Biologists under the terms of the Creative Commons Attribution License (https://creativecommons.org/licenses/by/4.0/).

Reviewer 3 Comments for the Author...

The manuscript is very well written, and it is easy to follow, however there are several points that should be addressed by the authors:

Introduction:
- The authors should avoid abbreviations if the full name is not described at least once in the text. Please correct accordingly throughout the introduction.

We would like to thank the reviewer for pointing this out. Abbreviations have now been used only when the full name is used previously in the text.

Methods:
- A paragraph should be included to explain the statistical analysis used in the manuscript.

A paragraph about statistical analysis is now included.

Results and figure legends:
- High magnification images from figures 1, 2 and 3 should be changed, the authors should show a 100X magnification image from a confocal microscope.

To do this would have meant repeating all the experiments so instead we have now included 2- or 4-x digital ZOOM images from the Zeiss microscope in new images in Figures 1 and 6 which clearly show Cx43 and Dlg1 at the plasma membrane in epidermis and in HaCaT cells.

- Figure 1C. When the authors show the results from the PLA, it seems that Cx43 and Dig 1 are localized at the nuclei, however images from Figure 1B do not show the same localization. Can the authors explain that?

We agree with the reviewer that the PLA images were not optimal. Since reviewer 1 did not believe that PLA allows one to conclude that two proteins directly interact, we have reverted to traditional confocal immunofluorescence microscopy. In the new Figure 1 we show Cx43 and Dlg1 colocalisation data from fresh cutaneous tissue. Cx43 and Dlg1 are largely co-located at the cell membrane.

- Western-blot from Figure 2D demonstrates that the higher Cx43 levels are present in HEK293 and NIKS, however these data do not correlate with the IF images showed in Figure 2A. Please explain. Also, why the authors decided to work with HaCaT cells if they have the lower Cx43 levels according to the western-blot in Figure 2D?

Immunofluorescence images are not visually quantitative and so it is hard to correlate with western blot data. We decided to continue to work with HaCaT cells because they can be grown relatively easily, compared to NIKS cells which require a complex cell culture system, and there is some controversy about the embryonic origin type of HEK293 cells that make them a less good model for keratinocytes.

- Statistics should be added to Figure 2E.

We didn't carry out statistics to compare the levels of Cx43 and Dlg1 between cell lines because these are all quite different types of cells. HaCaTs are spontaneously immortalised keratinocytes, NIKS are nearer to primary cells and require specialist complex cell culture conditions while HEK293 cells are not keratinocytes. To emphasise that we did not wish to compare levels of proteins between the cell types we have now split the original graph into three separate graphs (Figure 2B).

- What happen with Cx43 in Dig1-IP experiments in Figure 2G?

We have now included all the back blots and the reverse CoIP experiments in the new Figure 2

© 2023. Published by The Company of Biologists under the terms of the Creative Commons Attribution License (https://creativecommons.org/licenses/by/4.0/).

- Molecular weight should be added to western-blot n Figure 3C.

We would like to thank the reviewer for pointing out this error, which has now been corrected.

- Figure 4 should be revised by the authors. Figure legend does nor correlate with the data presented.

Figure 4 is now Figure 5 and we have revised the figure legend.

- HaCaT cells treated with NH4Cl, increase Dig 1 and Cx43 protein levels after 4 and 8 hours incubation (Fig 5C). These data are not shown in Mock and siRNa control in Figure 5C. Can the authors explain this fact?

This experiment was designed to determine the normal route of Cx43 degradation in HaCaT cells, not what happens when Dlg1 is depleted. The data showing what happens when Dlg1 is depleted is shown in the new Figure 6C (formerly Figure 5D).

- Number of experiments and number of replicates/experiments should be added in each figure legend.

We thank the reviewer for this important suggestion. We have added the number of replicates of each experiment to the Figure legends.

<u>Second decision letter</u>

MS ID#: JOCES/2022/259984

MS TITLE: The human Discs large protein controls Connexin 43 trafficking and gap junctional communication in keratinocytes.

AUTHORS: Harry Scott, Li Dong, Sharmila Srinivasan, Andrew Stevenson, Alasdair I MacDonald, Paola Massimi, Lawrence Banks, Patricia E Martin, Scott R Johnstone, and Sheila V Graham
ARTICLE TYPE: Research Article

We have now reached a decision on the above manuscript.

To see the reviewers' reports and a copy of this decision letter, please go to: https://submit-jcs.biologists.org and click on the 'Manuscripts with Decisions' queue in the Author Area. (Corresponding author only has access to reviews.)

As you will see, the reviewers gave favourable reports but raised some critical points that will require amendments to your manuscript. I hope that you will be able to carry these out because I would like to be able to accept your paper, depending on further comments from reviewers.

Please ensure that you clearly highlight all changes made in the revised manuscript. Please avoid using 'Tracked changes' in Word files as these are lost in PDF conversion.

I should be grateful if you would also provide a point-by-point response detailing how you have dealt with the points raised by the reviewers in the 'Response to Reviewers' box. Please attend to all of the reviewers' comments. If you do not agree with any of their criticisms or suggestions please explain clearly why this is so.

© 2023. Published by The Company of Biologists under the terms of the Creative Commons Attribution License (https://creativecommons.org/licenses/by/4.0/).

Reviewer 1

Advance summary and potential significance to field

This manuscript by Scott and colleagues investigates the functional role of the previously reported interaction between the scaffolding protein, human discs large protein (Dlg1) and Cx43 in previously uninvestigated keratinocytes. The authors provide evidence that this protein-protein interaction occurs in keratinocytes and epithelial tissue. They use Dlg1 knockdown strategies to show that this does not alter the transcript levels of Cx43 but leads to a reduction in Cx43, an increase in Cx43 levels in the Golgi apparatus, and an overall reduction in gap junction intercellular communication as revealed by dye transfer. The authors provide data supporting a mechanism where Dlg1 facilitates Cx43 trafficking and assembly into gap junctions in keratinocytes.

Comments for the author

Generally the manuscript has improved but a few lingering issues still exist.

1) The supplementary data as presented is not convincing. Maybe this is due to the PDF format but the EE1 labeling in Supplementary Figure 2 is not even evident and not reflective of early endosomes. This should be removed, redone or represented. Supplementary Figure 1 is okay but not great.
2) The Cx43 labeling in the adipocytes shown in Figure 1E looks odd and diffused. Are the authors convinced this is all specific?
3) The calnexin labeling looks different after siDlg knockdown in Figure 4. Also the Cx43 labeling is present in some cells and absence in others. Can the authors elaborate on what might be happening here?
4) Labeling for the 58K in control and siDlg cells also looks different and rather diffused for a Golgi marker (Figure 4G, H).
5) The LAMP2 labeling in Figure 6D, E is difficult to see and evidence of colocalization is not clear. This should be more clearly shown.
6) The quantification method of dye transfer is not clear as the authors refer to dye transfer to at least 200 cells. Please claifiy in the figure legend how the normalization was done and why reference is not made to either distance or average number of cells receiving dye from a donor cell. It is also not clear whether the HeLa cells are both the donor and receptor cells or whether the authors are assessing dye transfer between two different cell types.
7) In the model figure, the authors do not provide experimental evidence that Cx43 is subject to proteosomal degradation so not sure why there is an arrow suggesting that mode of degradation. Also, does the authors mean “late endosomes” when showing the fate of connexosomes as there are many types of endosomes to consider and possibly other modes of Cx43 internalization and degradation.

Reviewer 2

Advance summary and potential significance to field

This is a revision of a manuscript that describes interaction of Dlg1 with Cx43 not only in tumor cells (previous study) but also non-tumor cells. While the authors made many experimental changes and additions and significantly increased the quality of the data and of the manuscript, conclusions are still confusing and inconclusive.

Comments for the author

This is a revision of a manuscript that describes interaction of Dlg1 with Cx43 not only in tumor cells (previous study) but also non-tumor cells. While the authors made many experimental changes and additions, and significantly increased the quality of the data and of the manuscript, conclusions are still confusing and inconclusive. Two major concerns remain:

First, in the title, abstract, introduction, and other sections the authors state that Dlg1 interacts with Cx43 in keratinocytes. However, in the introduction they also say, “that the Dlg1-

© 2023. Published by The Company of Biologists under the terms of the Creative Commons Attribution License (https://creativecommons.org/licenses/by/4.0/).

Cx43 interaction occurs in a range of cell types in non-cancerous human cutaneous tissue and in cultured keratinocytes". This is confirmed by the results presented in Figure 1D/E and the authors write "Some colocalization was also present in some cells in the dermis (white arrowheads) suggesting that Cx43 interacts with Dlg1 in cells other than epithelial cells. The tissue sections also contained adipose tissue (**Figure 1E**) where colocalization of Cx43 with Dlg1 was apparent at cell membranes. These data indicate that Cx43 co-localizes with Dlg1 in several different cell types in cutaneous tissue." So, apparently, Cx43/Dlg1 co-localization is not limited to keratinocytes, but occurs in other tissues/cell types as well. Would it not be better to add this to title, abstract, etc., or alternatively leave cell type out? ("The human Discs large protein interacts with Connexin 43 in keratinocytes as well as other cell types" or "The human Discs large protein interacts with Connexin 43 in cancerous and non- cancerous tissues".)

Second, while the data presented convincingly demonstrate a Cx43/Dlg1 interaction, the authors conclude from their data that Dlg1 regulates forward trafficking of Cx43 to the plasma membrane. However, **stabilization of Cx43 gap junctions in the plasma membrane by Dlg1** (as observed for microtubule and actin interactions) **appears much more likely and is supported by the authors' data.** In Figure 3, the authors show that in Dlg1 knockdowns, total Cx43 levels go down (2B), both in PM (2C) and intracellularly (2D). Apparently, in Dlg1 KDs, Cx43 is lost from the PM and internalized (perinuclear region) and partially degraded by a non-endosomal/lysosomal (autophagy?) pathway (Figure 4). So, a more appropriate conclusion and title might be: "The human Discs large protein stabilizes Connexin 43 in the plasma membrane in keratinocytes and other cell types". Analyzing where in the cell the Cx43/Dlg1 interaction occurs (ER, Golgi, PM) might clarify the role Dlg1 plays in Cx43 gap junction biosynthesis, and this should be clarified before a potentially wrong role for Dlg1/Cx43 interaction is published. Of course, both hypotheses will result in a reduced cell-cell communication as shown in Figure 7.

Additional minor points:

l. 56: Citations; add significant related work from the Leithe, Falk, Berthoud labs l.67: Citations; add Shaw et al., Lauf et al.

l. 73: Citations, add Hunter and Gourdie, Rhett et al.

l. 76: Giepmans, not Geipmans

l. 101: ... inhibit recycling to the PM...; do you mean "forward trafficking to the PM"? l. 110: Change to "DLG1 functionally interacts with Cx43 ..."

l. 118: "in vitro and in vivo" to conform with Abstract and data order presented in Figure 1 (A-C, in vitro, D-E, in vivo)

l. 183: there is no Figure 3E

l. 275ff: The added Discussion section is confusing and is not consistent with a reduction of forward trafficking of Cx43 to the PM (no increase in proteasomal degradation). Indeed, the data is more consistent with loss of GJs from the PM. Published data indicates that GJs are internalized and degraded by phagosomal/lysosomal pathways, not endosomal, or proteasomal pathways.

l. 316ff: ZO-1, according to the Gourdie lab regulates accrual of newly synthesized connexons to the gap junction plaque from the perinexus. Indeed, Dlg1 could play an opposite role, stabilize GJs in the PM after channels are handed over from ZO-1 (ZO-1 is only bound to GJ channels in the plaque periphery) to Dlg1.

l. 400ff: section not justified

The Discussion, in its present format is much more a speculation. Acquiring some additional data on the function of DLg1 in GJ cell biology would reduce this issue.

© 2023. Published by The Company of Biologists under the terms of the Creative Commons Attribution License (https://creativecommons.org/licenses/by/4.0/).

Reviewer 3

Advance summary and potential significance to field

The authors demonstrates that the loss of Dlg1 reduces Cx43 at the plasma membrane accompanied by a reduction in the capacity of cells to communicate through gap junctions formed by Cx43.

Comments for the author

The authors have address all the suggestions. The manuscript is now suitable for publication

---

Second revision

Author response to reviewers' comments

Reviewer 1 Advance Summary and Potential Significance to Field:
This manuscript by Scott and colleagues investigates the functional role of the previously reported interaction between the scaffolding protein, human discs large protein (Dlg1) and Cx43 in previously uninvestigated keratinocytes. The authors provide evidence that this protein-protein interaction occurs in keratinocytes and epithelial tissue. They use Dlg1 knockdown strategies to show that this does not alter the transcript levels of Cx43 but leads to a reduction in Cx43, an increase in Cx43 levels in the Golgi apparatus, and an overall reduction in gap junction intercellular communication as revealed by dye transfer. The authors provide data supporting a mechanism where Dlg1 facilitates Cx43 trafficking and assembly into gap junctions in keratinocytes.

We would like to thank the reviewer for this nice summary of the work and recognition of the new inputs in the revised manuscript.

Reviewer 1 Comments for the Author:
Generally the manuscript has improved but a few lingering issues still exist.

1)The supplementary data as presented is not convincing. Maybe this is due to the PDF format but the EE1 labeling in Supplementary Figure 2 is not even evident and not reflective of early endosomes. This should be removed, redone or represented. Supplementary Figure 1 is okay but not great.

We had problems uploading figures to the JCS submission portal due to their size and so each figure was created as a PDF by the Editorial Office then re-PDFed to form the submitted manuscript. This decreased the quality of the images. We have reduced images sizes in the new resubmission to avoid this issue. However, we agree that the EEA1 data in Supplementary Figure 2 is not optimal. Since the endosomal data is not central to our conclusions, we have removed these data. We have improved the quality of Supplementary Figure 1 (now Fig. S2) by choosing different images.

2)The Cx43 labeling in the adipocytes shown in Figure 1E looks odd and diffused. Are the authors convinced this is all specific?

We have repeated the adipose tissue staining and reduced the brightness on the images. To make the images clearer, we have delineated the fat deposits with thin dotted white lines and shown an arrowhead in the 2x zoom image to indicate concentrated Dlg1/Cx43 colocalisation at the plasma membrane. The staining is specific since an IgG isotype negative control shows minimal staining and we have inserted a new panel in Fig. 1E. to show this.

3)The calnexin labeling looks different after siDlg knockdown in Figure 4. Also the Cx43 labeling is present in some cells and absence in others. Can the authors elaborate on what might be happening here?

© 2023. Published by The Company of Biologists under the terms of the Creative Commons Attribution License (https://creativecommons.org/licenses/by/4.0/).

Dlg1 depletion flattens cell shape (Rivera et al, Dev. Dyn https://www.ncbi.nlm.nih.gov/pmc/articles/PMC3016059/) giving rise to differences in the way the cells appear in confocal immunofluorescence microscopy and it is possible that changes in cell shape due to depletion of Dlg1 could alter the ER. We have mentioned this on page 8 line 207. We have improved Figure 4 by selecting more representative images where ER staining is not greatly altered between control and siDlg1-treated cells. These issues do not alter our conclusions that in Dlg1 depleted cells, Cx43 is intracellular but does not accumulate in the ER.

Our cells are transiently transfected so depletion levels will be different in individual cells. This is why the Cx43 labeling is present in some cells and reduced or absent in others.

4)Labeling for the 58K in control and siDlg cells also looks different and rather diffused for a Golgi marker (Figure 4G, H).

We used 58K and Golgi Tracker to visualise the Golgi and both gave very similar data. However, we agree that the original images shown were not ideal, particularly due to the double PDF conversion. They were chosen because of the strength of signal from the antibodies in the images. These have been replaced with more representative images. In differentiated keratinocytes (we cultured HaCaT cells under conditions in which some differentiation would occur) Golgi staining is known to become more diffuse Mahanty et al. Cell Death Differentiation: Keratinocyte differentiation promotes ER stress-dependent lysosome biogenesis - PubMed (nih.gov)). An explanation has been inserted on page 8 line 224.

5)The LAMP2 labeling in Figure 6D, E is difficult to see and evidence of colocalization is not clear. This should be more clearly shown.

The aim of this experiment was to demonstrate that very little Cx43 co-localised with LAMP2. The double PDF conversion has reduced the quality of the images. We have improved these now and included every channel and zoomed images with arrowheads indicating limited co-localisation. Like the Golgi, lysosomal staining becomes more diffuse when keratinocytes are differentiated Mahanty et al. Cell Death Differentiation: Keratinocyte differentiation promotes ER stress-dependent lysosome biogenesis - PubMed (nih.gov)). We have added a sentence to explain this on page 9 line 256.

6)The quantification method of dye transfer is not clear as the authors refer to dye transfer to at least 200 cells. Please claifiy in the figure legend how the normalization was done and why reference is not made to either distance or average number of cells receiving dye from a donor cell. It is also not clear whether the HeLa cells are both the donor and receptor cells or whether the authors are assessing dye transfer between two different cell types.

We already stated in the text that all acceptor cells were normal HaCaT cells. To make this clearer, we have added more explanation in the text in the Materials and Methods page 17 line 511 and in the Results section page 9 line 271. We have also included more detail in the figure legend.

7)In the model figure, the authors do not provide experimental evidence that Cx43 is subject to proteosomal degradation so not sure why there is an arrow suggesting that mode of degradation. Also, does the authors mean “late endosomes” when showing the fate of connexosomes as there are many types of endosomes to consider and possibly other modes of Cx43 internalization and degradation.

Cx43 is well known to be degraded by the proteasome. Therefore, we wished to include that mode of degradation in our model for completeness. However, we have now modified Figure 8 to take account of the important comment 2 of reviewer 2 and the model now focuses on delivery and maintenance of Cx43 at the membrane.

Reviewer 2

This is a revision of a manuscript that describes interaction of Dlg1 with Cx43 not only in tumor cells (previous study) but also non-tumor cells. While the authors made many experimental changes

© 2023. Published by The Company of Biologists under the terms of the Creative Commons Attribution License (https://creativecommons.org/licenses/by/4.0/).

and additions, and significantly increased the quality of the data and of the manuscript, conclusions are still confusing and inconclusive. Two major concerns remain:

First, in the title, abstract, introduction, and other sections the authors state that Dlg1 interacts with Cx43 in keratinocytes. However, in the introduction they also say, "that the Dlg1-Cx43 interaction occurs in a range of cell types in non-cancerous human cutaneous tissue and in cultured keratinocytes". This is confirmed by the results presented in Figure 1D/E and the authors write "Some colocalization was also present in some cells in the dermis (white arrowheads) suggesting that Cx43 interacts with Dlg1 in cells other than epithelial cells. The tissue sections also contained adipose tissue (**Figure 1E**) where colocalization of Cx43 with Dlg1 was apparent at cell membranes. These data indicate that Cx43 co-localizes with Dlg1 in several different cell types in cutaneous tissue." So, apparently, Cx43/Dlg1 co-localization is not limited to keratinocytes, but occurs in other tissues/cell types as well. Would it not be better to add this to title, abstract, etc., or alternatively leave cell type out? ("The human Discs large protein interacts with Connexin 43 in keratinocytes as well as other cell types" or "The human Discs large protein interacts with Connexin 43 in cancerous and non- cancerous tissues".)

We would like to thank the reviewer for this helpful analysis. We have changed the title and the text of the manuscript accordingly on page 11 line 285-289.

Second, while the data presented convincingly demonstrate a Cx43/Dlg1 interaction, the authors conclude from their data that Dlg1 regulates forward trafficking of Cx43 to the plasma membrane. However, **stabilization of Cx43 gap junctions in the plasma membrane by Dlg1** (as observed for microtubule and actin interactions) **appears much more likely and is supported by the authors' data**. In Figure 3, the authors show that in Dlg1 knockdowns, total Cx43 levels go down (2B), both in PM (2C) and intracellularly (2D). Apparently, in Dlg1 KDs, Cx43 is lost from the PM and internalized (perinuclear region) and partially degraded by a non-endosomal/lysosomal (autophagy?) pathway (Figure 4). So, a more appropriate conclusion and title might be: "The human Discs large protein stabilizes Connexin 43 in the plasma membrane in keratinocytes and other cell types".

The reviewer raises a very interesting and important point, for which we are very grateful. On checking our data, we now think this hypothesis may be correct. We apologise that we missed this possibility. Although there is much evidence to support a role for Dlg1 in forward trafficking of cargo proteins to the plasma membrane, Dlg1 is an adherens junction protein and so may well be involved in localising hemichannel delivery to the plasma membrane, especially as it is also known to interact indirectly with the actin cytoskeleton and microtubules. Thus, the mode of action of Dlg1 may be similar to Drebin in the control of Cx43. There is a good example of back tracking from the plasma membrane to the Golgi in the case of E-cadherin upon depletion of Scribble, an adherens junction PDZ protein whose function is related to Dlg1. We have altered the Abstract (page 2 line 54), the Introduction (page 5 line 132) and the Discussion (page 11/12 lines 310-320 and page 12 lines 326-355 to take into account this new hypothesis. We have also modified Figure 8 to include it in our model.

Analyzing where in the cell the Cx43/Dlg1 interaction occurs (ER, Golgi, PM) might clarify the role Dlg1 plays in Cx43 gap junction biosynthesis, and this should be clarified before a potentially wrong role for Dlg1/Cx43 interaction is published. Of course, both hypotheses will result in a reduced cell-cell communication as shown in Figure 7.

We believe we have demonstrated in this manuscript and in previous work that Cx43 interacts with Dlg1 mainly at the plasma membrane in non-tumour cells. In this manuscript we show that depletion of Dlg1 results in a level of loss of plasma membrane Cx43 very similar to levels of accumulation with markers of the Golgi but under these conditions residual Dlg1 remains on the membrane while Cx43 co-locates with markers of the Golgi (Figure 3). Quantification of Cx43 and Dlg1 co-location in different compartments of the cytoplasm in control and Dlg1 siRNA-treated cells is difficult due to low levels of the Dlg1 protein in these compartments, especially upon depletion.

Additional minor points:
l. 56: Citations; add significant related work from the Leithe, Falk, Berthoud labs

© 2023. Published by The Company of Biologists under the terms of the Creative Commons Attribution License (https://creativecommons.org/licenses/by/4.0/).

l.67: Citations; add Shaw et al., Lauf et al.

l. 73: Citations, add Hunter and Gourdie, Rhett et al.

l. 76: Giepmans, not Geipmans

l. 101: ... inhibit recycling to the PM...; do you mean “forward trafficking to the PM”?

l. 110: Change to “DLG1 functionally interacts with Cx43 ...”

l. 118: “in vitro and in vivo” to conform with Abstract and data order presented in Figure 1 (A-C, in vitro, D-E, in vivo)

l. 183: there is no Figure 3E

We apologise for missing out these important references which was due to swapping Endnote libraries from home office to work office. We have added the addition references and changed the text to deal with these minor points.

l. 275ff: The added Discussion section is confusing and is not consistent with a reduction of forward trafficking of Cx43 to the PM (no increase in proteasomal degradation). Indeed, the data is more consistent with loss of GJs from the PM. Published data indicates that GJs are internalized and degraded by phagosomal/lysosomal pathways, not endosomal, or proteasomal pathways.

We have changed the discussion on pages 11 and 12 (red text) to attempt to clarify this confusion.

l. 316ff: ZO-1, according to the Gourdie lab regulates accrual of newly synthesized connexons to the gap junction plaque from the perinexus. Indeed, Dlg1 could play an opposite role, stabilize GJs in the PM after channels are handed over from ZO-1 (ZO-1 is only bound to GJ channels in the plaque periphery) to Dlg1.

We have now mentioned that Dlg1 could stabilise gap junctions in the Discussion on page 12 line 319.

l. 400ff: section not justified

The Discussion, in its present format is much more a speculation. Acquiring some additional data on the function of DLg1 in GJ cell biology would reduce this issue.

We believe that our study is sound as it stands, as indicated by the other reviewers. We agree that the Discussion is speculative in parts. We felt this was important to give the reader an indication of our future experimental plans as regards the function of Dlg1. We hope we have now removed much of the speculation from the Discussion.

Reviewer 3
Advance Summary and Potential Significance to Field:
The authors demonstrates that the loss of Dlg1 reduces Cx43 at the plasma membrane accompanied by a reduction in the capacity of cells to communicate through gap junctions formed by Cx43.

Reviewer 3 Comments for the Author:
The authors have address all the suggestions. The manuscript is now suitable for publication.

We would like to thank the reviewer for this positive review and thank them for their efforts.

© 2023. Published by The Company of Biologists under the terms of the Creative Commons Attribution License (https://creativecommons.org/licenses/by/4.0/).

Third decision letter

MS ID#: JOCES/2022/259984

MS TITLE: The human Discs large protein (Dlg1) is required for gap junctional communication.

AUTHORS: Harry Scott, Li Dong, Sharmila Srinivasan, Andrew Stevenson, Alasdair I MacDonald, Paola Massimi, Lawrence Banks, Patricia E Martin, Scott R Johnstone, and Sheila V Graham
ARTICLE TYPE: Research Article

We have now reached a decision on the above manuscript.

To see the reviewers' reports and a copy of this decision letter, please go to: https://submit-jcs.biologists.org and click on the 'Manuscripts with Decisions' queue in the Author Area. (Corresponding author only has access to reviews.)

As you will see, two of the referees continue to raise critical points that will require amendments to your manuscript. Both referees feel that the title is overstated. In addition, referee #2 has several additional concerns that all need to be addressed prior to acceptance.

Given that you have had several opportunities to revise, this will be the final opportunity to completely address the referees concerns, and there is no guarantee of acceptance. Depending on your response (and also revised manuscript with changes clearly highlighted), I may have referee #2 look at the revised manuscript.

Please ensure that you clearly highlight all changes made in the revised manuscript. Please avoid using 'Tracked changes' in Word files as these are lost in PDF conversion.

I should be grateful if you would also provide a point-by-point response detailing how you have dealt with the points raised by the reviewers in the 'Response to Reviewers' box. Please attend to all of the reviewers' comments. If you do not agree with any of their criticisms or suggestions please explain clearly why this is so.

Reviewer 1

Advance summary and potential significance to field

See past submission

Comments for the author

This is my third time assessing this manuscript and it continues to improve. I only have two remaining concerns.

1) The title has changed in this last version and is now not accurate as written and highly overstated. There are lots of connexins involved in GJIC so a title that indicates Dlg1 is required for intercellular communication is highly overstated given the enclosed data.
2) The y axis in Figure 7E has compressed font that looks poor.

Reviewer 2

Advance summary and potential significance to field

In this revision the authors further improved the quality of the manuscript, however several points need further revision before publication, in part due to a rather superficial revision.

© 2023. Published by The Company of Biologists under the terms of the Creative Commons Attribution License (https://creativecommons.org/licenses/by/4.0/).

Comments for the author

In response to the reviewer, the authors changed their interpretation of the role of Dlg1 from playing a role in forward trafficking to a role in stabilizing GJs in the plasma membrane. This clearly says something about the robustness of the presented data. In addition, the authors changed the title from "The human Discs large protein (Dlg1) controls Connexin 43 (Cx43) trafficking to the plasma membrane and gap junctional communication in keratinocytes" to "The human Discs large protein (Dlg1) is required for gap junctional communication". The chosen new title is an extremely stark statement that is not sufficiently supported by the results. Are the authors convinced Dlg-1 is required for the formation and maintenance of Cx43-based gap junctions in all cells that make Cx43 GJs? This is what the new title implies. Indeed, loss of GJs from the PM upon Dlg1 KD shown by IF and Westerns (Fig. 2 and 3) is rather moderate; clearly nowhere close to 100%. A title such as "Dlg1 interacts with and stabilizes Cx43 gap junctions in the plasma membrane" that is more in line with the new interpretation of the results seems to be safer at this early stage of characterization of DLG1's role in Cx43 GJ function. The authors should really think about the robustness of their data.

Results, L. 288: "… data strongly suggest … by facilitating trafficking of Cx43 to the plasma membrane." This is conflicting with the statement in the Discussion, L. 338: "suggesting that Dlg1 could be involved in trafficking of Cx43 to the plasma membrane. However, this is unlikely since we found no evidence of Dlg1 colocalisation with Cx43 at intracellular sites (Fig. 3)." Loss of GJs from the PM, of course, will also result in reduced GJIC as shown in Fig. 7. The concluding statement in the Results section should be changed to "maintaining Cx43 GJs in the plasma membrane" which would be consistent with the rest of the manuscript.

Figure 8: Are the authors implying connexons can "diffuse" through tight junctions. Is there published evidence for that? Maybe Fig. 8 needs to be re-drawn.

Abstract, l. 49: Add "also" or "in addition" for clarity as authors previously showed Dlg1/Cx43 interaction in tumor cells.

L. 205 and throughout: "membrane-associated" is unclear (which membrane). Do not use "membrane", if "plasma membrane" is meant as even intracellular Cxs are membrane associated.

L. 212: Fig. S2, not S1

L. 270/271: Fig. 6F not 6E

L. 304: "formation"; more appropriate would be "mainenance" or "function"

L. 316: "intercellular cellular communication"

L. 350: "bind a [the] terminal five aminoacids of Cx43"

L. 98: Giepmans is still misspelled.

<u>Reviewer 3</u>

Advance summary and potential significance to field

-

Comments for the author

The authors have addressed all the questions. I think the manuscript should be accepted for publication without any further changes required from the authors

© 2023. Published by The Company of Biologists under the terms of the Creative Commons Attribution License (https://creativecommons.org/licenses/by/4.0/).

Third revision

Author response to reviewers' comments

Reviewer 1 Advance Summary and Potential Significance to Field:
See past submission

Reviewer 1 Comments for the Author:
This is my third time assessing this manuscript and it continues to improve. I only have two remaining concerns.

We are glad the reviewer believes we have improved our manuscript and we thank them for their patient reviews.

1) The title has changed in this last version and is now not accurate as written and highly overstated. There are lots of connexins involved in GJIC so a title that indicates Dlg1 is required for intercellular communication is highly overstated given the enclosed data.

We agree entirely that we have altered the title so that it is no longer accurate, and we apologise for this error.
We were aiming to keep the title within the 120 character limit but have gone too far. We propose a new title based on a previous suggestion of Reviewer 2, which we hope is more accurate:

The human Discs large protein (Dlg1) interacts with and maintains Connexin 43 at the plasma membrane in keratinocytes.

2) The y axis in Figure 7E has compressed font that looks poor.

We have corrected the Y-axis in Figure 7E.

Reviewer 2 Advance Summary and Potential Significance to Field:
In this revision the authors further improved the quality of the manuscript, however several points need further revision before publication, in part due to a rather superficial revision.

In our revised manuscript, in response to reviewer 1 we modified Figures 1, 4, 6 and 8, including adding additional images/data, to improve the overall data. Reviewer 1 now agrees that we have done so. Moreover, in response to this reviewer we altered our hypotheses and conclusions and amended/added sections to the manuscript throughout. We would argue that these changes are more than a superficial revision.

Reviewer 2 Comments for the Author:
In response to the reviewer, the authors changed their interpretation of the role of Dlg1 from playing a role in forward trafficking to a role in stabilizing GJs in the plasma membrane. This clearly says something about the robustness of the presented data.

We believe our data are robust. We have shown clearly, as commented by this reviewer in their previous review that “the data presented convincingly demonstrate a Cx43/Dlg1 interaction”. We have shown that a 73% siRNA depletion of Dlg1 in keratinocytes leads to a 72% reduction in Cx43 protein levels and that the remaining Cx43 locates to the Golgi. Finally, and importantly, we show that a 73% Dlg1 depletion results in a 55% reduction in gap junctional communication in a parachute assay. In comparison, chemical blockage of gap junctional communication by 50 μM carbenoxolone results in a 65% reduction. This strongly suggests that Dlg1 makes an important contribution to gap junctional communication.

In terms of data interpretation, there is good evidence for a role of Dlg1 in trafficking of other proteins to the plasma membrane, as we point out in our Discussion. There is no evidence so far of Dlg1 acting to stabilise membrane proteins, although a Dlg1-related protein Scribble, has been shown to do so. Therefore, it is possible for there to be more than one explanation for the effect of Dlg1 depletion on Cx43 levels and activity. It would take a further three years of work, or more, to distinguish between Dlg1 involvement in forward trafficking versus Dlg1 acting to stabilize Cx43 at

© 2023. Published by The Company of Biologists under the terms of the Creative Commons Attribution License (https://creativecommons.org/licenses/by/4.0/).

the plasma membrane and we would prefer to progress publishing the data we have at present. Given the role of ZO-1 and drebrin in stabilising connexons at the plasma membrane, it seems prudent to preferentially propose this hypothesis at this time.

In addition, the authors changed the title from “The human Discs large protein (Dlg1) controls Connexin 43 (Cx43) trafficking to the plasma membrane and gap junctional communication in keratinocytes” to “The human Discs large protein (Dlg1) is required for gap junctional communication”. The chosen new title is an extremely stark statement that is not sufficiently supported by the results. Are the authors convinced Dlg-1 is required for the formation and maintenance of Cx43-based gap junctions in all cells that make Cx43 GJs? This is what the new title implies. Indeed, loss of GJs from the PM upon Dlg 1 KD shown by IF and Westerns (Fig. 2 and 3) is rather moderate; clearly nowhere close to 100%. A title such as “Dlg 1 interacts with and stabilizes Cx43 gap junctions in the plasma membrane” that is more in line with the new interpretation of the results seems to be safer at this early stage of characterization of DLGI's role in Cx43 GJ function. The authors should really think about the robustness of their data.

We are not sure if the reviewer has made a mistake in quoting the figure numbers. Figure 2 does not show any data with Dlg1 knock down. Perhaps the reviewer means Figures 3 and 4? In Figure 3 we show that a 73% reduction in Dlg 1 levels results in a 72% reduction in Cx43 levels, which we would argue is more than “moderate”. The reviewer is correct that loss of Cx43 from the plasma membrane is rather moderate in the images in Figure 4. However, the reason for this is that we designed the experiments such that we depleted Dlg1 by only 42% so that we could still visualise Cx43 in the cells. When we depleted Dlg1 by >70% we could no longer quantify Cx43 in our images. Therefore, changes in Cx43 subcellular localisation in Figure 4 are indeed moderate due to a moderate reduction in Dlg 1 levels. As listed above, we believe our data are sufficiently robust, particularly given the results of our parachute assay which strongly suggest that Dlg 1 makes an important contribution to gap junctional communication.

As in our response to Reviewer 1, we would like to apologise for creating an unsuitable title. Our new title takes account of this reviewer’s suggestion:

The human Discs large protein (Dlg1) interacts with and maintains Connexin 43 at the plasma membrane in keratinocytes.

Results, L. 288: "... data strongly suggest ... by facilitating trafficking of Cx43 to the plasma membrane.” This is conflicting with the statement in the Discussion, L. 338: "suggesting that Dlg 1 could be involved in trafficking of Cx43 to the plasma membrane. However, this is unlikely since we found no evidence of Dlg 1 colocalisation with Cx43 at intracellular sites (Fig. 3).” Loss of GJs from the PM, of course, will also result in reduced GJIC as shown in Fig. 7. The concluding statement in the Results section should be changed to "maintaining Cx43 GJs in the plasma membrane” which would be consistent with the rest of the manuscript.

We agree with the reviewer and would like to thank them for pointing out this inconsistency. We have changed the concluding statement in the Results section.

Figure 8: Are the authors implying connexons can "diffuse” through tight junctions. Is there published evidence for that? Maybe Fig. 8 needs to be re-drawn.

We are fully aware that gap junctions do not diffuse through tight junctions and it was never our intention to illustrate such a concept. It is well established that the ‘junctional nexus’ comprised of gap junctions, tight and adherens junctions, localise in similar areas of the plasma membrane and have aspects in common with adapter and linker proteins enabling interaction with the cytoskeleton. We have modified Figure 8 to maintain focus on connexins and gap junctions.

Abstract, l. 49: Add "also” or "in addition” for clarity as authors previously showed Dlg1/Cx43 interaction in tumor cells.

The word “also” has been inserted.

© 2023. Published by The Company of Biologists under the terms of the Creative Commons Attribution License (https://creativecommons.org/licenses/by/4.0/).

L. 205 and throughout: "membrane-associated” is unclear (which membrane). Do not use "membrane”, if "plasma membrane” is meant as even intracellular Cxs are membrane associated.

Thank you for pointing this out. This has been changed throughout the manuscript.

L. 212: Fig. S2, not S1. We have changed this number.

L. 270/271: Fig. 6F not 6E. We don't have a figure 6E.
L. 304: "formation”; more appropriate would be "mainenance” or "function”. We have changed "formation” to "function”

L. 316: "intercellular cellular communication”

L. 350: "bind a [the] terminal five aminoacids of Cx43”

L. 98: Giepmans is still misspelled.

We have corrected these errors and thank the reviewer for their careful reading of our manuscript.

Reviewer 3 Advance Summary and Potential Significance to Field:

Reviewer 3 Comments for the Author:
The authors have addressed all the questions. I think the manuscript should be accepted for publication without any further changes required from the authors

We are glad that his reviewer suggests that the manuscript is now suitable for publication.

Fourth decision letter

MS ID#: JOCES/2022/259984

MS TITLE: The human Discs large protein (Dlg1) interacts with and maintains Connexin 43 at the plasma membrane in keratinocytes.

AUTHORS: Harry Scott, Li Dong, Sharmila Srinivasan, Andrew Stevenson, Alasdair I MacDonald, Paola Massimi, Lawrence Banks, Patricia E Martin, Scott R Johnstone, and Sheila V Graham
ARTICLE TYPE: Research Article

I am happy to tell you that your manuscript has been accepted for publication in Journal of Cell Science, pending standard ethics checks. I did want to raise one other point, which I should have mentioned before. That is to ensure that you are doing your statistics on the average of biological repeats, not the technical repeats (for instance not on cell-cell borders of cells within a single coverslip). I am asking this because some of your p values are less than I would expect. (More significant). We recommend that you use superplots to then show the spread of data amongst the technical repeats. Let us know if you have questions about this, and if you require another revision that would be acceptable.

© 2023. Published by The Company of Biologists under the terms of the Creative Commons Attribution License (https://creativecommons.org/licenses/by/4.0/).
